# Synthesis, characterization and application of oxovanadium(iv) complexes with [NNO] donor ligands: X-ray structures of their corresponding dioxovanadium(v) complexes†

**DOI:** 10.1039/d2ra01448c

**Published:** 2022-05-06

**Authors:** Rakhimoni Borah, Surabhi Lahkar, Naranarayan Deori, Sanfaori Brahma

**Affiliations:** Department of Chemistry, Gauhati University Guwahati 781014 Assam India sanfaoribrahma@gmail.com

## Abstract

Two oxovanadium(iv) complexes ligated by [NNO] donor ligands have been synthesized and characterized by ESI-HRMS, elemental (CHN) analysis and spectroscopic (UV-Vis, IR and EPR) techniques. Block shaped brown crystals from the methanolic solutions of these oxovanadium(iv) complexes were obtained during the crystallization process. Crystallographic structures of the resulting crystals revealed that the original oxovanadium(iv) complexes have been transformed into new dioxovanadium(v) complexes with concomitant oxidation of V^IV^ to V^V^. The original oxovanadium(iv) complexes have been identified to be an efficient catalyst for the CO_2_ cycloaddition reaction with epoxides resulting up to 100% cyclic carbonate products. The geometries of oxovanadium(iv) complexes are optimized by the density functional theory (DFT) calculations at the uB3LYP/6-31G**/LANL2DZ level of theory. The geometry and structural parameters of optimized structures of oxovanadium(iv) complexes are in excellent agreement with the parameters of X-ray structures of their dioxovanadium(v) counterparts. Further, TD-DFT and Spin Density Plots for the oxovanadium(iv) complexes are performed in order to get more insights about their electronic absorption and EPR spectroscopies, respectively.

## Abstract 2

Oxovanadium(iv) complexes catalysed CO_2_ cycloaddition resulting up to 100% conversion of epoxides to cyclic carbonates under relatively benign condition. Transformation of oxovanadium(iv) to dioxovanadium(v) in the process of crystallization.

## Graphical abstract

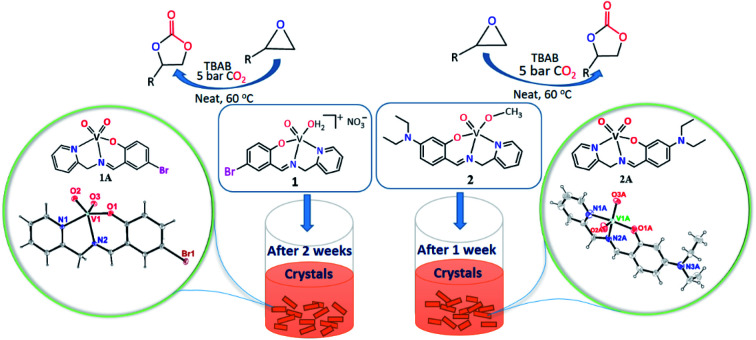

## 1 Introduction

Carbon dioxide, a major greenhouse gas, is being released into the Earth's atmosphere in a large scale due to the combustion of fossil fuels leading to global warming, which is at present posing a serious environmental threat.^1–5^ Nevertheless, fossil fuel burning is still inevitable due to the increasing demand of energy and lack of feasible technologies for sustainable energies.^1^ Therefore, the strategies that need to be immediately adopted are the reduction in carbon dioxide emission and the attenuation of carbon dioxide concentration in the atmosphere. On the other hand, CO_2_ is an abundant, renewable, inexpensive and non-toxic C1 source of chemical carbon in organic syntheses.^1–15^ Although utilization of carbon dioxide as C1 feedstock is challenging in view of its thermodynamic and kinetic inertness,^16^ research that deals with sustainable use of CO_2_ in producing value-added chemicals is advancing at a rapid rate.^17–21^ Of many important CO_2_ transformation reactions,^22^ cycloaddition of CO_2_ with epoxides to form cyclic carbonates stands out to be a very attractive transformation process.^23–57^ The process is 100% atom economic and the resulting cyclic carbonate products are commercially important chemicals which can be used as electrolytes in lithium-ion battery,^58^ polar aprotic solvents,^59^ functionalized building blocks for synthesizing valuable organic products,^60^ monomers for polycarbonates^61^ and isocyanate-free polyurethanes,^62^ intermediates for pharmaceuticals and fine chemicals.^63^ Interestingly, cyclic carbonate moieties are also seen in some natural products^64–66^ thereby making the CO_2_ transformation reaction to cyclic carbonate a very significant and naturally relevant reaction.

Catalytic cycloaddition of CO_2_ with epoxides to yield cyclic carbonates using both homogeneous and heterogeneous catalyst systems that encompass metal complexes,^31–39,50–56^ ionic liquids,^40,41^ metal organic frameworks (MOFs),^42,57^ covalent organic frameworks (COFs),^43^ porous organic polymers (POPs),^44^ organo-catalysts,^45^ bi-functional catalysts,^46^ alkali metal salts,^47,48^ and metal oxides^49^ have been demonstrated by various research groups. Currently, vanadium complexes have gained considerable attention from scientific community to be used as catalyst in the CO_2_ cycloaddition with epoxides due to the Lewis acidic nature of high-valent V^V^ and V^IV^ centers, suitable for activating epoxides as well as relatively non-toxic nature of naturally abundant vanadium metal.^50–57^ Lee and co-workers^67^ were first to report commercial VCl_3_ catalysed cycloaddition reaction of CO_2_ with epoxides, however it required high temperature (90–120 °C) and pressure (14.8 bar).

Herein, we report synthesis, characterization and the catalytic study of substituted salicylidin-2-picolylimine ligated vanadium complexes in the CO_2_ cycloaddition with epoxides yielding cyclic carbonates with very good to excellent conversions under relatively benign conditions.

## 2 Results and discussion

### 2.1 Syntheses and structures of the vanadium complexes

**Scheme 1:**
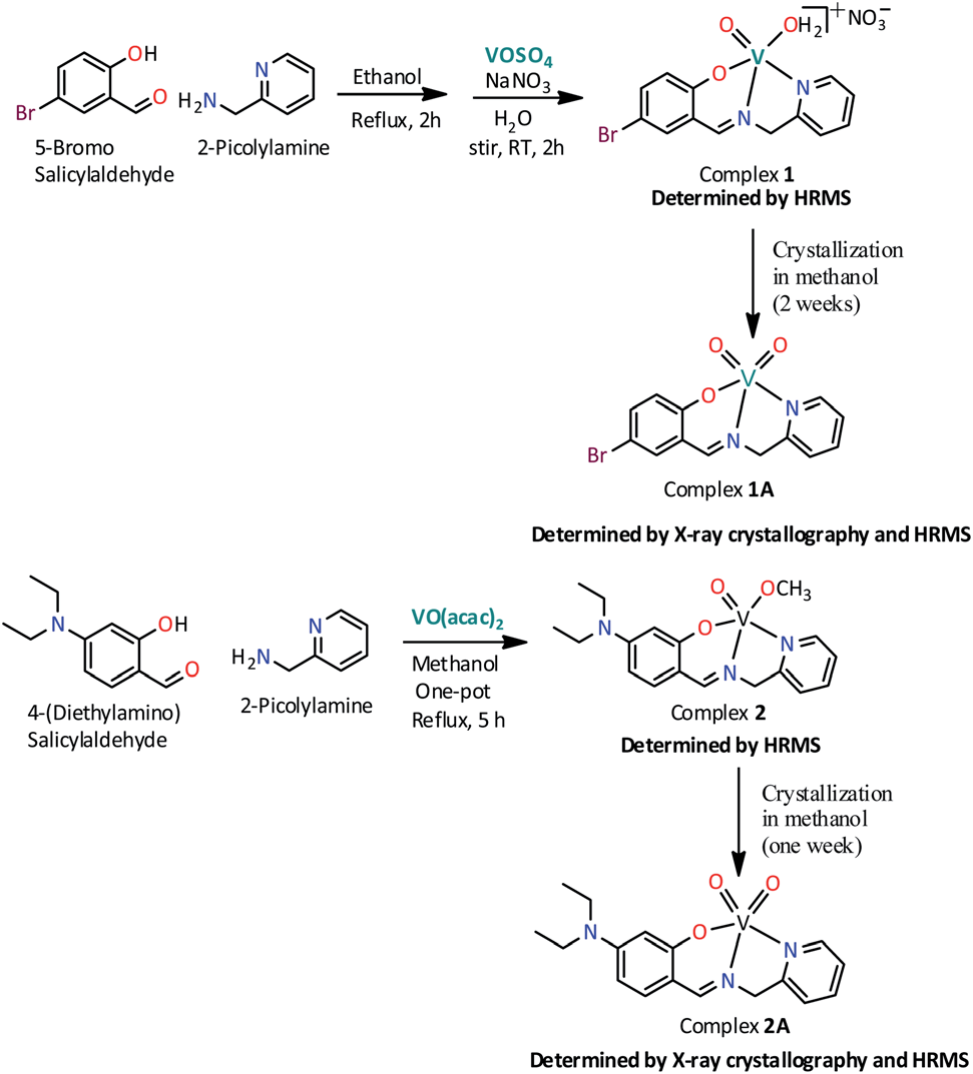
Synthetic pathway for the preparation of complexes 1, [V^IV^O(H_2_O)L1]NO_3_; 1A, [V^V^O_2_L1]; 2, [V^IV^O(OCH_3_)L2] and 2A, [V^V^O_2_L2]. (HL1 = 5-bromosalicylidin-2-picolylimine; HL2 = 4-diethylaminosalicylidin-2-picolylimine).

Scheme 1 illustrates the synthetic route for two new oxovanadium complexes 1, [V^IV^O(H_2_O)L1]NO_3_ and 2, [V^IV^O(OCH_3_)L2] and their corresponding dioxovanadium complexes 1A, [V^V^O_2_L1] and 2A, [V^V^O_2_L2] (HL1: 5-bromosalicylidin-2-picolylimine; HL2: 4-diethylaminosalicylidin-2-picolylimine).

Complex 1 was prepared by first refluxing an equimolar mixture of 5-bromosalicylaldehyde and 2-picolylamine in ethanol for 2 h, followed by addition of aqueous VOSO_4_ solution and subsequent refluxing for another 1 h. Green solid complex 1 was obtained when an aqueous solution of sodium nitrate was added to the former solution and stirred for 1 h at room temperature. Dioxovanadium(v) complex, 1A was obtained as brown blocked shaped crystals from the methanolic solution of complex 1 after around two weeks. Similarly, complex 2 has been synthesized in one pot by refluxing equimolar mixture of 4-(diethylamino)salicylaldehyde and 2-picolylamine in methanol for 2 h and subsequently refluxing further for 3 h after addition of 1 equivalent [VO(acac)_2_]. The solution obtained was filtered and from the filtrate greenish color solid is collected and dried. Dioxovanadium(v) complex, 2A was obtained as brown blocked shaped crystals from the methanolic solution of complex 2 after around one week.

### 2.2 X-ray crystallography

**Fig. 1:**
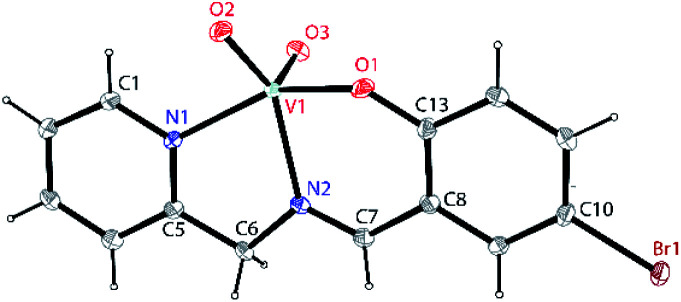
A perspective view of the asymmetric unit of complex 1A, [V^V^O_2_L1] (HL1 = 5-bromosalicylidin-2-picolylimine) showing 50% thermal contours for all non-hydrogen atoms at 273(2) K. H-atoms are shown as small spheres with arbitrary radii.

Single crystals suitable for X-ray crystallographic analysis of dioxovanadium(v) complexes 1A, [V^V^O_2_L1] and 2A, [V^V^O_2_L2] were obtained from their corresponding oxovanadium(iv) complexes 1, [V^IV^O(H_2_O)L1]NO_3_ and 2, [V^IV^O(OCH_3_)L2]. However, crystals of complexes 1 and 2 could not be obtained in spite of repeated attempts. Brown block shaped crystals of 1A suitable for X-ray diffraction were obtained *via* slow evaporation of solution containing complex 1 in methanol for around 2 weeks. Complex 1A crystallises in a monoclinic crystal system with *P*2_1_/*c* space group. Perspective view of the asymmetric unit of complex 1A and the crystal packing are illustrated in Fig. 1 and Fig. S1 (ESI†), respectively. Crystal data collection parameters and the selected bond distances and angles are shown in Table S1 and S2 (ESI†), respectively. V(1)–O(2), 1.637(2) Å and V(1)–O(3), 1.620(2) Å bond lengths are in accordance with the usual V=O bond distances.^68^ V(1)–N(1) and V(1)–N(2) bond lengths are comparable which are 2.115(3) and 2.153(3) Å, respectively. However, V(1)–O(1) bond length, 1.900(2) Å is significantly smaller than V–N bond distances. C(6)–N(2) and C(7)–N(2) bond distances, 1.475(4) Å and 1.293(4) Å are consistent with the C–N and C=N bond distances. Moreover, C(10)–Br(1) bond distance, 1.901(3) Å is consistent with C–Br bond distances. O(2)–V(1)–O(3) bond angle is in line with the previously reported values 109.32(12)°.

**Fig. 2:**
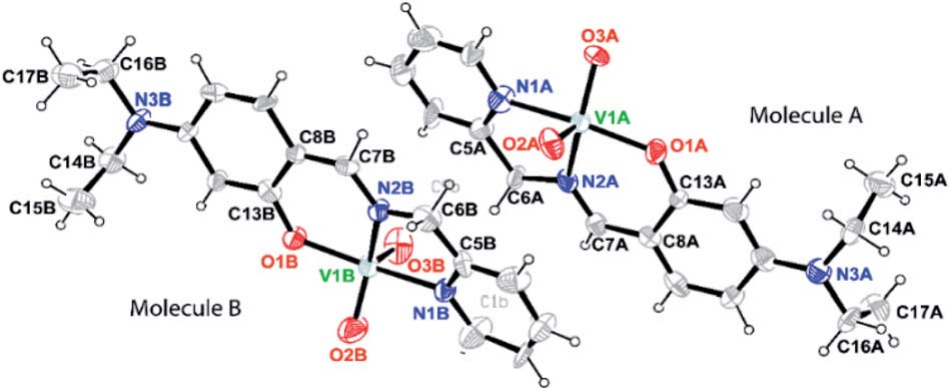
A perspective view of the asymmetric unit of complex 2A, [V^V^O_2_L2] (HL2 = 4-diethylaminosalicylidin-2-picolylimine) showing 50% thermal contours for all non-hydrogen atoms at 296(2) K. H-atoms are shown as small spheres with arbitrary radii.

Brown block shaped crystals of 2A were grown *via* slow evaporation of solution containing complex 2 in methanol for around 7 days. Complex 2A crystallises in a monoclinic crystal system with *P*2_1_ space group. Perspective view of the asymmetric unit of complex 2A and the crystal packing are illustrated in Fig. 2 and S2 (ESI†), respectively. Asymmetric unit of the crystal contains two symmetry-independent molecules *viz*A and B. It is observed that molecules are crystallographically non-equivalent as the corresponding bond distances and angles differ slightly in these two molecules (Table S3, ESI†). The V–N bond distances in molecule A is somewhat shorter than those in molecule B [V(1A)–N(1A), 2.136(5) Å and V(1B)–N(1B), 2.140(5) Å; V(1A)–N(2A), 2.097(5) Å and V(1B)–N(2B), 2.127(4) Å in molecule A and B, respectively]. However, the V–O bond distance in molecule A is somewhat longer compared to that in molecule B, V(1A)–O(1A), 1.910(4) Å and V(1B)–O(1B), 1.888(3) Å. It is worthy to mention that the bond distances C(6A)–N(2A), 1.504(7) Å and C(6B)–N(2B), 1.434(8) Å in molecules A and B, respectively significantly differ. Moreover, both the V=O bond distances in molecule A [V(1A)–O(2A), 1.638(6) Å; V(1A)–O(3A), 1.654(5) Å] is comparatively longer compared to those in molecule B [V(1B)–O(2B), 1.609(6) Å; V(1B)–O(3B), 1.595(6) Å].

### 2.3 Structure optimization of [1 – NO_3_^−^]^+^ (without NO_3_^−^ counter anion) and 2

**Fig. 3:**
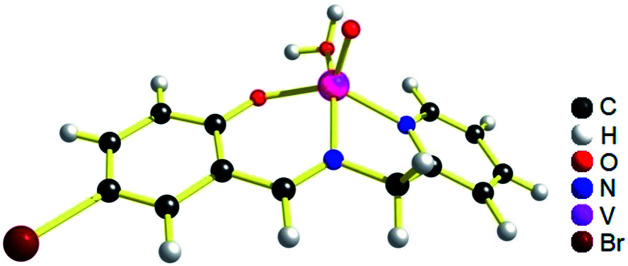
uB3LYP/LANL2DZ/6-31G** optimized structure of complex [1 – NO_3_^−^]^+^ (without NO_3_^−^ counter anion).

**Fig. 4:**
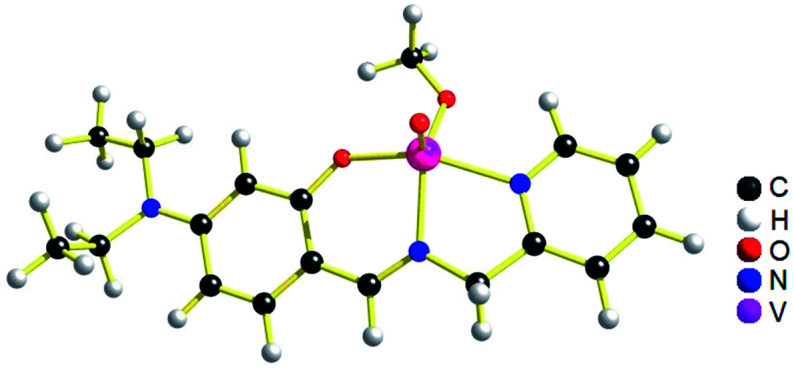
uB3LYP/LANL2DZ/6-31G** optimized structure of complex 2, [V^IV^O(OCH_3_)L2].

The geometries of [1 – NO_3_^−^]^+^ and 2 are optimized by the density functional theory (DFT) calculations at the uB3LYP/6-31G**/LANL2DZ level of theory. The geometry and structural parameters of complexes [1 – NO_3_^−^]^+^ and 2 determined by DFT calculations are in excellent agreement with the parameters of X-ray structures of their dioxovanadium(v) counterparts. In both the complexes, the coordination geometry around vanadium(iv) center is square pyramidal with oxo ligands in the axial position (Fig. 3 and 4). Excluding the bonds, V–O(H_2_O) and V–O(OCH_3_) which are respectively absent in 1A and 2A, the selected bond distances of the optimised structures of both the complexes [1 – NO_3_^−^]^+^ and 2 (Table 1) are similar with the experimentally determined X-ray structures of their dioxovanadium(v) counterparts, 1A and 2A.

**Table 1:** Selected bond lengths in angstroms of UB3LYP-optimized structures of [1 – NO_3_^−^]^+^ and 2

Bond lengths (Å)	[1 – NO_3_^−^]^+^	2
V=O	1.582	1.601
V–O (H_2_O)	2.134	—
V–O (OCH_3_)	—	1.854
V–O (phenolate)	1.879	1.946
V–N (Py)	2.103	2.168
V–N (imine)	2.026	2.093
C=N	1.302	1.308
C–Br	1.953	—

### 2.4 NMR spectroscopy

In spite of repeated attempts, no good ^1^H NMR spectrum of complex 1 was obtained, may be due to the presence of paramagnetic vanadium(iv) center.^69^ However, ^1^H NMR spectrum of complex 2 is reasonably good (Fig. S3†) although the signals are broad. The resonance at 8.80 ppm is due to the imine (–C*H*=N) proton. There appear four signals (8.51, 7.98, 7.61 and 7.44 ppm) for picolyl aromatic protons. The resonance at 5.33 ppm can be attributed to the picolyl methylene proton. Moreover, the phenolate proton signals appear at 7.22, 6.20 and 5.90 ppm. It is worth mentioning that –C*H*_2_ protons of ethyl groups and –OC*H*_3_ protons resonate in the same region with residual water impurity.

### 2.5 EPR spectroscopy

**Fig. 5:**
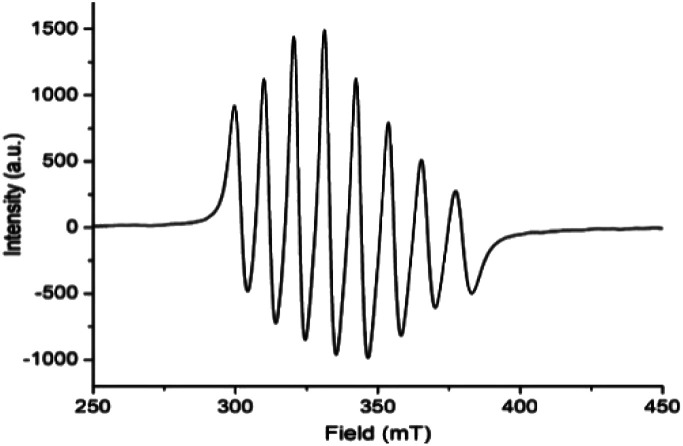
Room temperature X-band EPR spectrum of complex 1, [V^IV^O(H_2_O)L1]NO_3_ (HL1 = 5-bromosalicylidin-2-picolylimine) in chloroform.

**Fig. 6:**
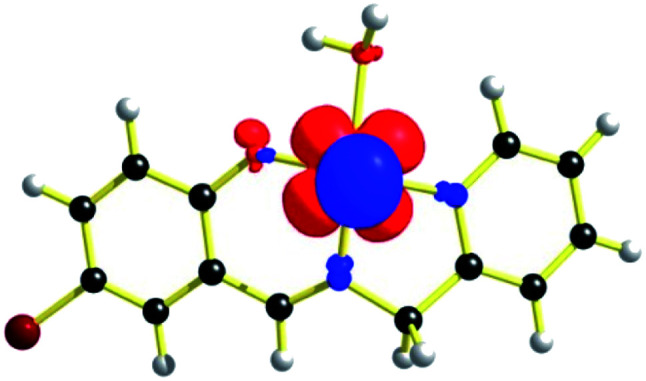
Spin density plot of [1 – NO_3_^−^]^+^ (iso value: 0.004) from DFT (uB3LYP level).

EPR spectrum (Fig. 5) of complex 1, [V^IV^O(H_2_O)L1]NO_3_ in chloroform exhibited eight-lines revealing the oxidation state of vanadium as +4. The hyperfine structure corresponds to the interaction of a single unpaired electron (*S* = 1/2) with a vanadium nucleus (*I* = 7/2). The typical eight-line pattern X-band EPR spectrum of the complex 1 (*g*_iso_ = 1.9905, *A*_iso_ = 96.695 G) recorded in room temperature reveals the presence of a single vanadium(IV) center in the complex.^70^ Spin density plot of the complex [1 – NO_3_^−^]^+^ (without NO_3_^−^ counter anion) shown in Fig. 6 reveals the localisation of single spin (*S* = 1/2) on vanadium(IV) center with no spin delocalisation over ligand, thus supporting the experimental EPR signal.^71,72^

**Fig. 7:**
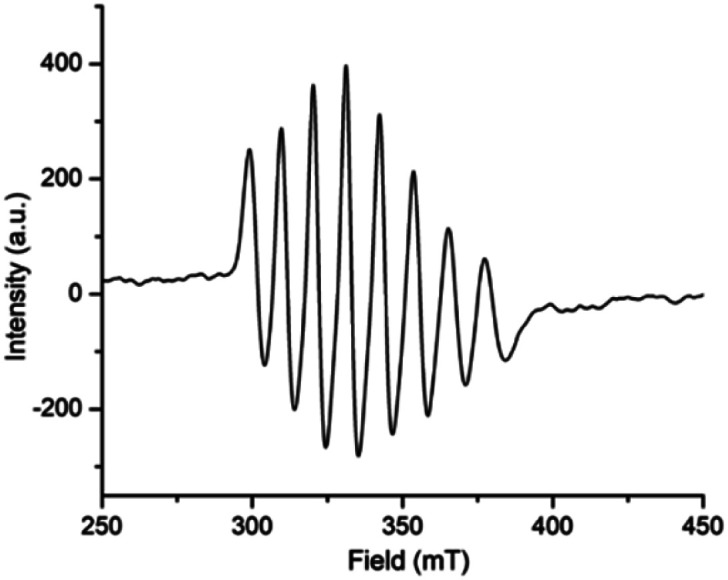
Room temperature X-band EPR spectrum of complex 2, [V^IV^O(OCH_3_)L2] (HL2 = 4-diethylaminosalicylidin-2-picolylimine) in methanol.

**Fig. 8:**
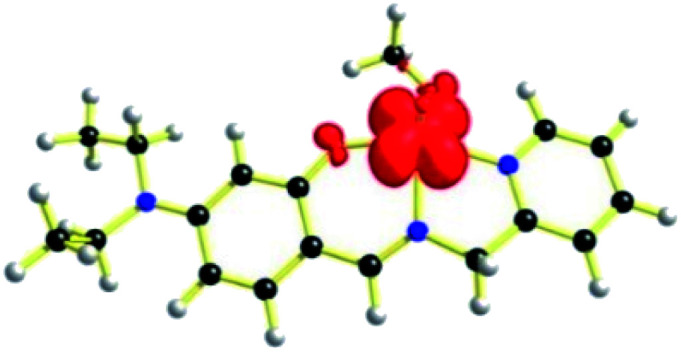
Spin density plot of complex 2 (iso value: 0.004) from DFT (uB3LYP level).

Expectedly, similar kind of EPR spectrum (Fig. 7) is also observed for the oxovanadium(iv) complex 2 (*g*_iso_ = 1.9935, *A*_iso_ = 96.557 G). Moreover, the spin density plot of the complex 2 (Fig. 8) also indicates the localisation of spin density on oxovanadium(iv) center like that in complex [1 – NO_3_^−^]^+^.

### 2.6 IR spectroscopy

The IR spectra of oxovanadium(iv) complex 1, [V^IV^O(H_2_O)L1]NO_3_ and complex 2, [V^IV^O(OCH_3_)L2] are shown in Fig. S4 and S5 (ESI†), respectively. The IR spectrum of oxovanadium complex 1 displayed a strong band at 964 cm^−1^ that can be attributed to *ν*(V=O) of the vanadyl moiety present in the complex.^73^ Moreover, the peak at 1627 cm^−1^ is related to vibration of the azomethine moiety (C=N).^74^ Further the peaks at 1761, 1383 and 830 cm^−1^ are attributable to the NO_3_^−^.^75^ In a similar way, the IR spectrum of oxovanadium complex 2 reveals the formation of the complex which shows a peak at 924 cm^−1^ corresponding to *ν*(V=O) stretching of the vanadyl group. Further the strong band observed at 1596 cm^−1^ corresponds to *ν*(C=N) of the azomethine group.^76^

### 2.7 UV-visible spectroscopy

**Fig. 9:**
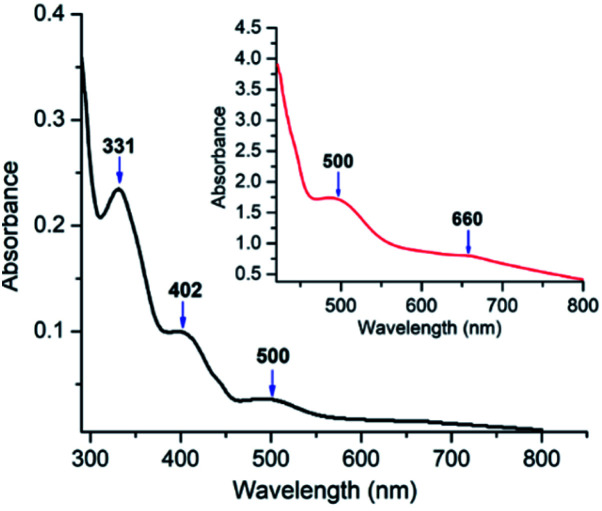
UV-visible spectra in dichloromethane of complex 1 (5 × 10^−5^ M) at 298 K. Inset represents UV-visible spectrum of 1 (3 × 10^−3^ M) in dichloromethane.

The UV-visible spectrum of oxovanadium(iv) complex 1 (5.0 × 10^−5^ M) in dichloromethane have been illustrated in Fig. 9. UV-visible spectrum of 1 displays a peak in the higher energy region at 331 nm, which is due to ligand centred transitions.^77^ Band appearing at 402 nm is attributed to the LMCT transitions originating from the p orbital of phenolate oxygen to the empty d orbital of vanadium(iv) center.^78^ Moreover, d–d transition in complex 1 is apparent (500 and 660 nm) when the concentration of 1 was increased to 3.0 × 10^−3^ M.^79^ Similarly, UV-visible spectrum of 2 (Fig. S6,† left) exhibits a band at 349 nm corresponding to π–π* transition of azomethine chromophore.^80,81^ The intense band observed at 402 nm can be attributed to the charge transfer transition (LMCT) from the p orbital of the phenolate oxygen to empty d orbital of vanadium(iv).^78^ However, the bands due to d–d transitions could not be distinguished properly as they are probably under the tail of much stronger LMCT band at *ca.* 465 nm (shoulder).^82^ Moreover, the UV-visible spectrum (Fig. S6,† right) recorded in DMF solvent of dioxovanadium(v) complex 2A shows similar absorption pattern showing LMCT bands at 396 and 465 nm (shoulder) as that of oxovanadium(iv) complex 2, suggesting that complex 2 get oxidised to complex 2A.

**Fig. 10:**
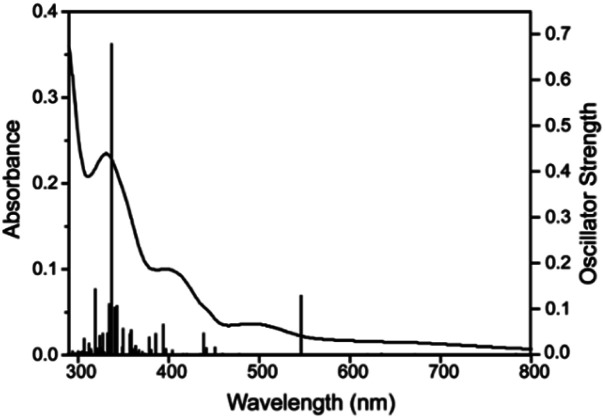
UV-visible spectrum in dichloromethane (curved line, left axis) and oscillator strengths (vertical line, right axis) obtained from TD-DFT calculations at the CAM-B3LYP/6-31G**/LANL2DZ level of theory for [1 – NO_3_^−^]^+^.

The time-dependent density functional theory (TD-DFT) calculation of [1 – NO_3_^−^]^+^ shown in Fig. 10 indicates the presence of absorption band at longer wavelength (546 nm; oscillator strength *f* = 0.1281) that can be attributable to the d–d transition.

### 2.8 ESI-HRMS spectrometry

The formation of complex 1, [V^IV^O(H_2_O)L1]NO_3_ and 2, [V^IV^O(OCH_3_)L2] were further unambiguously confirmed by the electrospray ionization high-resolution mass spectrometry (ESI-HRMS). A peak appearing at *m*/*z* 373.9460 in the HRMS (Fig. S7, ESI†) corresponds to [1 – NO_3_^−^]^+^ (calcd, 373.9471) and an additional peak at *m*/*z* 396.9604 corresponds to [1 − NO_3_^−^ + Na]^+^ (calcd, 396.9369). The other fragment ions observed in the spectrum at *m*/*z* 355.9344 and 386.9532 correspond to [1 − NO_3_^−^ − H_2_O]^+^ (calcd, 355.9365) and [1 − NO_3_^−^ − H_2_O + CH_3_OH]^+^ (calcd, 386.9549), respectively. Formation of complex 2 was also revealed by the ion peak at *m*/*z* 380.0787 (Fig. S8, ESI†) that corresponds to the [2]^+^ (calcd, 380.1179). Further, the transformation of complex 2, [V^IV^O(OCH_3_)L2] to 2A, [V^V^O_2_L2] during the course of crystallization process was also confirmed when the crystals of complex 2A were subjected to mass spectrometry that showed an ion peak at *m*/*z* 366.1039 corresponding to the [2A + H]^+^ (calcd, 366.1023) (Fig. S9, ESI†). The other peak observed in the mass spectrum of 2A at *m*/*z* 284.1775 (Fig. S9, ESI†) corresponds to the detached ligand HL2, 4-diethylaminosalicylidin-2-picolylimine (calcd, 284.1763).

### 2.9 Cycloaddition reaction of CO_2_ with epoxides

**Scheme 2:**
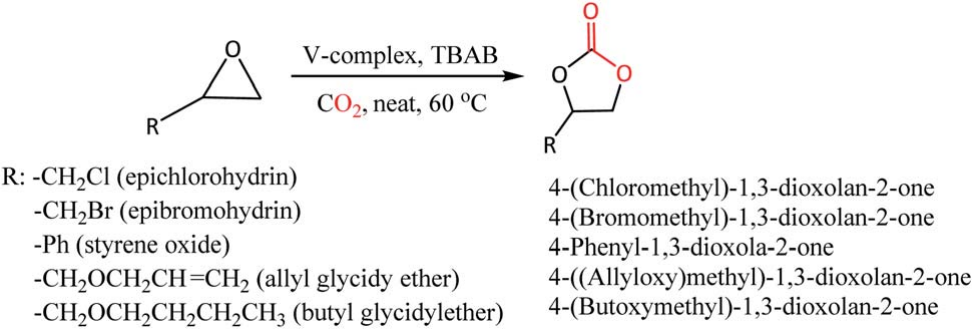
Catalytic cycloaddition reaction of CO_2_ with epoxides using oxovanadium(iv) complexes 1 and 2 in presence of co-catalyst TBAB.

CO_2_ cycloaddition with epoxides for the formation of cyclic carbonates was performed by loading vanadium(iv) complexes 1 or 2 with tetrabutylammonium bromide (TBAB) co-catalyst in a stainless-steel autoclave equipped with a magnetic stirring bar and dosing appropriate pressure of CO_2_ (Scheme 2, Table 2). The formation of cyclic carbonates has been confirmed by ^1^H NMR (Fig. S10–S22, ESI†) showing downfield shifting of the peaks compared to epoxides because of the addition of CO_2_. The formation of cyclic carbonate products was also supported by the IR spectroscopic technique, which showed characteristic peaks in a range of 1783–1786 cm^−1^ that correspond to *ν*(C=O) of carbonate moiety (Fig. S23–S25†). Preliminary investigation of the potentiality of the catalyst system 1-TBAB (1 mol%: 2 mol%) was performed at 60 °C and 5 bar CO_2_ pressure taking epichlorohydrin (ECH) and the epibromohydrin (EBH) as the substrates. To our delight, quantitative conversions of ECH (entry 1, 100% conv.) and EBH (entry 2, 100% conv.) to their corresponding cyclic organic carbonates (COCs) were achieved. To see the catalytic activity of the oxovanadium(iv) complex 1 alone, we have performed the reaction in the absence of TBAB co-catalyst (entry 3). However, it is revealed that vanadium complex 1 alone does not have any capability to transform the styrene oxide (SO) into its corresponding COC. To see the catalytic efficiency of the complex 2, a similar kind of reaction was performed taking ECH as substrate and found 2-TBAB catalyst system to have the same extent of efficiency (entry 4, 100% conv.) as that of 1-TBAB. To see the substrate scope of the catalyst systems, we have chosen three other substrates *viz.* styrene oxide (SO), allyl glycidyl ether (AGE) and butyl glycidyl ether (BGE). Both the catalyst systems 1-TBAB and 2-TBAB showed similar kinds of conversions when SO was taken as substrate (compare entry 5 and 6, 95% and 93% conv., respectively). Comparing the entry 5 (95% conv.) and entry 7 (26% conv.), it is observed that 1-TBAB catalyst system is superior to TBAB alone, as more than around 68% conversion was found when the combination of complex 1 and TBAB was used as a catalyst system instead of TBAB alone. Catalyst system 2-TBAB appears to be a bit better than 1-TBAB in case of the substrate AGE (compare entry 8 and 9, 85% and 92% conv., respectively). For the substrate BGE, both the vanadium complexes 1 and 2 showed similar potentiality as catalysts resulting in 85% (entry 10) and 82% (entry 11) conversions for catalyst 1 and 2, respectively. To investigate the effect of CO_2_ pressure on the catalytic activity of the catalyst system, a reaction was performed with SO as substrate under 1 bar of CO_2_ pressure keeping other parameters the same. It is seen that 100% conversion of SO (entry 12) to its corresponding COC could be achieved within 6 h of duration in a mere 1 bar of CO_2_ pressure indicating 1-TBAB combination to be a very efficient catalyst.

**Table 2:** CO_2_ cycloaddition reaction to epoxides using complexes 1 and 2^a^

Entry	Epoxide	Catalyst	1 or 2 (mol%)	TBAB (mol%)	Time (h)	% Conv^b^	Ref.
1	ECH	1-TBAB	1	2	4	100	Tw^c^
2	EBH	1-TBAB	1	2	4	100	Tw^c^
3	SO	1	1	—	4	0	Tw^c^
4	ECH	2-TBAB	1	2	4	100	Tw^c^
5	SO	1-TBAB	1	2	4	95	Tw^c^
6	SO	2-TBAB	1	2	4	93	Tw^c^
7	SO	TBAB	—	2	4	26	Tw^c^
8	AGE	1-TBAB	1	2	4	85	Tw^c^
9	AGE	2-TBAB	1	2	4	92	Tw^c^
10	BGE	1-TBAB	1	2	4	85	Tw^c^
11	BGE	2-TBAB	1	2	4	82	Tw^c^
12	SO	1-TBAB	1	2	6	100	Tw^d^
13	SO	TBAB	—	2	6	33	Tw^d^

a Reaction conditions: ECH = 0.5 mL (6.38 mmol); SO = 0.5 mL (4.38 mmol); AGE = 0.5 mL (4.27 mmol); BGE = 0.5 mL (3.50 mmol); EBH = 0.5 mL (5.88 mmol).

b Percentage conversions of the catalytic reactions were evaluated with the help of ^1^H NMR spectroscopy according to the conversion formula shown in Fig. S26.

c 5 bar CO_2_ pressure.

d 1 bar CO_2_ pressure.

### 2.10 Catalytic cycle

**Scheme 3:**
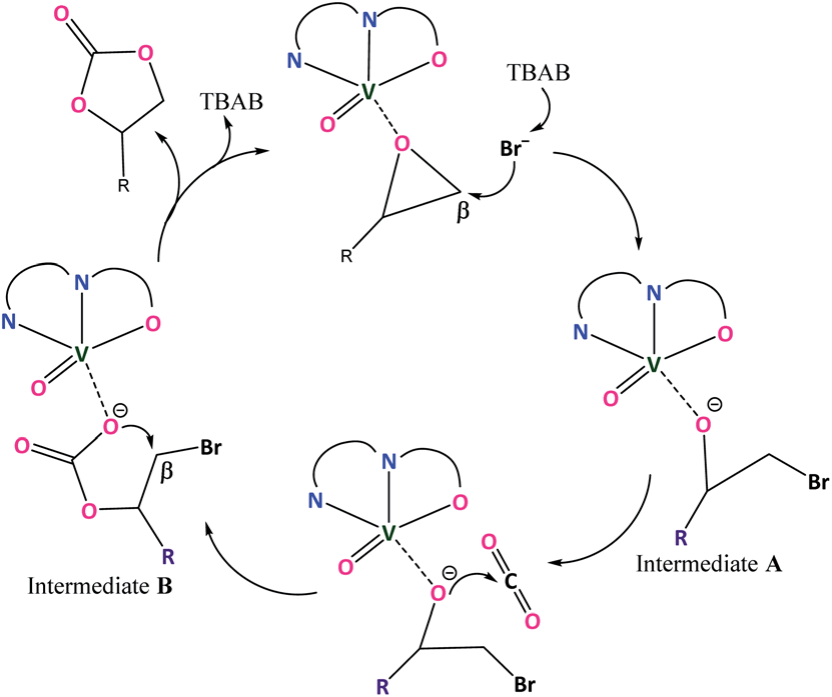
Plausible catalytic cycle.

As suggested from previously reported work^52^ on metal complex catalysed conversion of carbon dioxide and epoxides into cyclic carbonates, an attempt has been made to sketch a plausible reaction pathway for cycloaddition of carbon dioxide to epoxides catalysed by oxovanadium(iv) catalysts 1 and 2 (Scheme 3). Lewis acidic V(iv) center of oxovanadium(iv) complexes 1 and 2 activates the epoxide by coordinating through the epoxide O-atom which results in the weakening of β C–O bond as well as increase in the electrophilic nature of the β carbon of the epoxide. As a result, bromide anion from TBAB attacks epoxide β-carbon more facilely and cleaves the O–CH_2_ bond to produce intermediate A. In the next step, oxyanion intermediate A makes a nucleophilic attack at the carbon center of CO_2_ to generate a carbonate species (intermediate B). In the final step, the ring closure through nucleophilic attack by carbonate species at β-carbon results in the cyclic carbonate products.

## 3 Conclusion

Two new oxovanadium(iv) complexes 1, [V^IV^O(H_2_O)L1]NO_3_ and 2, [V^IV^O(OCH_3_)L2] with [NNO] donor ligand have been synthesized and characterized by spectroscopic techniques (UV-Vis, IR and EPR), ESI-HRMS and elemental (CHN) analysis. Block shaped brown crystals obtained from the methanolic solutions of complexes 1 and 2 were crystallographically characterised and revealed to be dioxovanadium complexes 1A, [V^V^O_2_L1] and 2A, [V^V^O_2_L2] respectively. Crystal structures determined for complexes 1A and 2A indirectly give an evidence for the formation of complexes 1 and 2 where the vanadium(iv) centers were slowly oxidised to vanadium(v) during the crystallization process. However, structures of oxovanadium(iv) complexes [1 − NO_3_^−^]^+^ and 2 have been optimized by the density functional theory (DFT) calculations at the uB3LYP/6-31G**/LANL2DZ level of theory. It is worthy to mention that the structural parameters of the optimized structures are in very good agreement with their dioxovanadium(v) counterparts, 1A and 2A. TD-DFT calculation for the complex 1 (without NO_3_^−^ anion) could support the presence of electronic absorption bands at longer wavelengths (546 nm, oscillator strength *f* = 0.1281) attributable to d–d transitions. Moreover, spin density plot of the oxovanadium(iv) complexes [1 − NO_3_^−^]^+^ and 2 show localization of spin density on the V(iv) centers supporting the experimental EPR lines. An investigation on the catalytic activity of the oxovanadium(iv) complexes in the cycloaddition reaction of carbon dioxide with the epoxides to yield cyclic organic carbonates as products revealed that V-complexes 1 and 2 are efficient catalysts. Applicability of dioxovanadium(v) complexes 1A and 2A as catalysts in various reactions are also being investigated.

## 4 Experimental and computational section

### 4.1 Materials

All chemicals used in this work were purchased from Sigma Aldrich, Spectrochem, and Alfa Aesar, and were used without further purification.

### 4.2 Instrumentation

IR spectra were recorded on a BRUKER infrared spectrometer (model no ALPHA II). Elemental analysis (CHN) measurements were done in Thermo Scientific FlashSmart CHNS/O analyzer. The electronic spectra were recorded using a Shimadzu UV-2600 spectrophotometer. ^1^H NMR spectra were recorded on Bruker 400 MHz, Bruker 500 MHz, Bruker 600 MHz, JEOL 400 MHz and JEOL 500 MHz spectrometers with tetramethyl silane (TMS) as an internal standard. The solvents used were CDCl_3_ and [D_6_] DMSO. Chemical shifts are reported in parts per million (*δ*) and spin multiplicities are indicated by the following symbols: s (singlet), d (doublet), t (triplet), m (multiplet) and dd (doublet of doublets). A WATERS XEVO-G2XSQTOF was used for taking mass spectra. The X-band electron paramagnetic resonance (EPR) spectra were recorded on a JESFA200 ESR spectrometer at room temperature with the experimental conditions [frequency, 9.439 GHz; power, 0.995 mW; field center, 490.000 mT; width, ± 500.000 mT; sweep time, 30.0 s; modulation frequency, 100 00 kHz; width, 1.0000 mT; amplitude, 100; and time constant, 0.03 s]. The crystals of complex 1 and 2 were coated with light hydrocarbon oil and mounted at 273(2) K and 296(2) K, respectively on a Bruker SMART APEX CCD diffractometer, and the intensity data were collected using graphite-monochromated Mo Kα radiation (*λ* = 0.71073 Å). The data integration and reduction were processed with SAINT software.^83^ An absorption correction was applied.^84^ The structure was solved by the direct method using SHELXT 2014/5. The structure was refined on *F*^2^ by the full-matrix least-squares technique using the SHELXL-2018/3 program package.^85^ Non-hydrogen atoms were refined anisotropically. In the refinement, hydrogens were treated as riding atoms using SHELXL default parameter.

#### 4.2.1 Computational details

Gaussian 09, revision B.01, program^86^ was used to carry out the DFT calculations and the method used was Becke's three-parameter hybrid-exchange functional,^87–89^ with the nonlocal correlation provided by the Lee, Yang, and Parr expression and the Vosko, Wilk, and Nuair 1980 correlation functional (III) for local correction.^90,91^ Geometry optimizations were executed in which all of the coordinates were taken from the molecular structures wherever possible. Geometry optimizations were carried out for both the complexes [1 − NO_3_^−^]^**+**^ and 2 using the basis set 6-31G** for the C, N, O and H atoms and LANL2DZ for the V atom while excluding the counter anion for the calculations. To confirm that the optimized geometries did not have imaginary frequencies, frequency calculations were performed. In order to consider the solvent effect (dichloromethane was used as the solvent), self-consistent reaction field method was applied in all of the calculations. Zero-point energies and thermal corrections were also included. As no imaginary frequencies were found, the optimized geometry was ensured to be the potential energy minima by vibrational frequency calculations at the same level of theory. The Chemcraft software program^92^ was used to visualize the orbital surfaces. All of the TD-DFT calculations were performed at the CAM-B3LYP level.

### 4.3 Synthesis of complexes

#### 4.3.1 Synthesis of complex 1, [V^IV^O(H_2_O)L1]NO_3_ (HL1 = 5-bromosalicylidin-2-picolylimine)

A solution prepared by dissolving 0.108 g (1 mmol) of 2-picolylamine in 10 mL ethanol was mixed with a solution of 0.201 g (1 mmol) of 5-bromosalicylaldehyde in 10 mL ethanol in a round bottom flask and heated under reflux for 2 h which results in a yellow solution. To this solution was added 0.163 g (1 mmol) of vanadyl sulphate monohydrate by dissolving in 20 mL of distilled water and stirred for another 1 h at room temperature. Then a solution of 0.085 g (1 mmol) sodium nitrate in 5 mL water was added and again stirred for another 1 h to get a green precipitate. The reaction mixture was then filtered and the precipitate obtained was washed with little amount of water and dried in a desiccator to produce green color solid of complex 1. Yield (0.281 g, 75%). Anal. calcd (found) for C_13_H_12_N_2_O_3_BrV [1 − NO_3_^−^]^+^: C, 41.63 (42.12); H, 3.22 (2.92); N, 7.47 (6.41). UV/Vis *λ*_max_ (dichloromethane)/nm 331 (*ε*/dm^3^ mol^−1^ cm^−1^ 4.70 × 10^3^), 402 (1.98 × 10^3^), 500 (5.70 × 10^4^) and 660 (2.66 × 10^4^). IR *ν*_max_/cm^−1^ 1761 (NO_3_^−^), 1627 (C=N), 1521, 1452, 1383 (NO_3_^−^), 1310, 1151, 964 (V=O), 830 (NO_3_^−^) and 710. ESI-HRMS(+) [M − NO_3_^−^]^+^: *m*/*z* (calcd) = 373.9471, *m*/*z* (found) = 373.9460.

#### 4.3.2 Synthesis of complex 2, [V^IV^O(OCH_3_)L2] (HL2 = 4-diethylaminosalicylidin-2-picolylimine)

A solution of 0.216 g (2 mmol) 2-picolylamine dissolved in 20 mL methanol was taken in a round bottom flask and added to it a solution of 0.386 g (2 mmol) 4-(diethylamino)salicylaldehyde in methanol dropwise. The contents of the flask were then refluxed with stirring for about 2 h and then a solution of VO(acac)_2_ (0.532 g, 2 mmol) in 25 mL methanol was added and the reaction mixture was further refluxed for another 3 h with continued stirring. After cooling to room temperature, the reaction mixture was filtered and evaporation of the filtrate in open air afforded greenish color solid of complex 2. Yield (0.315 g, 83%). Anal. calcd (found) for C_18_H_23_N_3_O_3_V: C, 56.84 (56.46); H, 6.10 (5.57); N, 11.05 (10.19). UV/Vis *λ*_max_ (dichloromethane)/nm 349 (ε/dm^3^ mol^−1^ cm^−1^ 9.1 × 10^3^), 402 (1.45 × 10^4^) and 465 (3.02 × 10^3^). IR *ν*_max_/cm^−1^ 1596 (C=N), 1507, 1392, 1346, 1248, 1141, 924 (V=O) and 772. ESI-HRMS(+) [M]^+^: *m*/*z* (calcd) = 380.1179, *m*/*z* (found) = 380.0787. ^1^H NMR *δ*_H_ (600 MHz, DMSO-*d*_6_) 8.80 (–C*H*=N, 1H), 8.51 (Picolyl py-*H*, 1H), 7.98 (Picolyl py-*H*, 1H), 7.61 (Picolyl py-*H*, 1H), 7.44 (Picolyl py-*H*, 1H), 7.22 (Ph-*H*, 1H), 6.20 (Ph-*H*, 1H), 5.90 (Ph-*H*, 1H), 5.33 (picolyl-C*H*_2_, 2H), 3.25 (ethyl-C*H*_2_, –OC*H*_3_, 7H), and 0.99 (–C*H*_3_, 6H).

### 4.4 General procedure for catalytic reactions yielding cyclic carbonates

The oxovanadium(iv) complex 1 or 2 (1 mol%), TBAB (2 mol%) and epoxide (0.5 mL) were mixed together in a 100 mL stainless steel autoclave and the existing air in the reaction vessel was evacuated with a flush of CO_2_ gas for some time. The reaction vessel was then pressurized with appropriate pressure of CO_2_ and stirred for desired time at 60 °C. Upon completion of the catalytic reaction, the residual CO_2_ of the autoclave was vented out carefully. After cooling the autoclave to room temperature, the reaction mixture was collected for analysis.

#### 4.4.1 4-(Chloromethyl)-1,3-dioxolan-2-one

^1^H NMR *δ*_H_ (500 MHz, CDCl_3_) 5.02 (m, 1H), 4.61 (dd, 1H), 4.41 (dd, 1H) and 3.82–3.77 (m, 2H). IR *ν*_max_/cm^−1^ 1785 (C=O), 1163 (C–O).

#### 4.4.2 4-(Bromomethyl)-1,3-dioxolan-2-one

^1^H NMR *δ*_H_ (400 MHz, CDCl_3_) 4.96 (m, 1H), 4.58 (dd, 1H), 4.33 (dd, 1H) and 3.58 (m, 2H).

#### 4.4.3 4-Phenyl-1,3-dioxolan-2-one

^1^H NMR *δ*_H_ (500 MHz, CDCl_3_) 7.39–7.33 (m, 5H), 5.63 (dd, 1H), 4.75 (dd, 1H) and 4.29 (dd, 1H). ^13^C NMR (100 MHz, CDCl_3_, ppm) *δ*: 155.33, 136.13, 129.77, 129.28, 126.04, 78.34, 71.31. IR *ν*_max_/cm^−1^ 1787 (C=O), 1161 (C–O).

#### 4.4.4 4-[(Allyloxy)methyl]-1,3-dioxolan-2-one

^1^H NMR *δ*_H_ (500 MHz, CDCl_3_) 5.83 (m, 1H), 5.23 (m, 2H), 4.79 (m, 1H), 4.48 (dd, 1H), 4.37 (dd, 1H), 4.03 (m, 2H), 3.66 (dd, 1H) and 3.58 (dd, 1H). IR *ν*_max_/cm^−1^ 1783 (C=O), 1167 (C–O).

#### 4.4.5 4-(Butoxymethyl)-1,3-dioxolan-2-one

^1^H NMR *δ*_H_ (500 MHz, CDCl_3_) 4.83 (m, 1H), 4.49 (dd, 1H), 4.38 (dd, 1H), 3.67 (dd, 1H), 3.60 (dd, 1H), 3.51 (t, 2H), 1.55 (m, 2H), 1.35 (m, 2H) and 0.91 (t, 3H).

## Author contributions

Rakhimoni Borah: Data curation, methodology, investigation, validation, visualization and writing - original draft. Surabhi Lahkar: Writing - review & editing. Naranarayan Deori: Software. Sanfaori Brahma: Conceptualization, funding acquisition, project administration, supervision and resources.

## Conflicts of interest

There are no conflicts to declare.

## Supplementary Material

RA-012-D2RA01448C-s001

RA-012-D2RA01448C-s002
