# Supplementary material for: Synthesis, characterization and application of oxovanadium(iv) complexes with [NNO] donor ligands: X-ray structures of their corresponding dioxovanadium(v) complexes
Source: RSC Adv. 2022 May 6;12(22):13740–8. doi: 10.1039/d2ra01448c (PMC9076100; doi:10.1039/d2ra01448c)
Supplement: RA-012-D2RA01448C-s001 [file RA-012-D2RA01448C-s001.pdf]

Electronic Supplementary Material (ESI) for RSC Advances.
This journal is © The Royal Society of Chemistry 2022

Synthesis, characterization and application of oxovanadium(IV) complexes with [NNO] donor ligands: X-ray structures of their corresponding dioxovanadium(V) complexes

Rakhimoni Borah, Surabhi Lahkar, Naranarayan Deori and Sanfaori Brahma*[a]

Table S1: Crystal data and data collection parameters for complex **1A, [V^VO_2L1]** (HL1 = 5-Bromosalicylidin-2-picolylimine) and complex **2A, [V^VO_2L2]** (HL2 = 4-Diethylaminosalicylidin-2-picolylimine).

Crystal data	Complex **1A**	Complex **2A**
Formula	$C_{13}H_{10}BrN_2O_3V$	$C_{17}H_{20}N_3O_3V$
T, (K)	273(2) K	296(2) K
Formula weight	373.07	365.30
Color	Brown	Brown
Crystal system	Monoclinic	Monoclinic
Space group	$P2_1/c$	$P2_1$
a, Å	7.9790(12)	6.6846(7)
b, Å	16.281(3)	8.3382(9)
c, Å	9.8619(14)	30.000(3)
α, deg	90°	90°
β, deg	98.160(4)°	90.978(3)°.
ϒ, deg	90°	90°
V, Å^3	1268.2(3)	1671.9(3)
Radiation (λ, Å)	Mo Kα (0.71073)	Mo Kα (0.71073)
Z	2	4
d_{calcd}, g.cm^{-3}	1.954	1.451
F(000)	736	760
μ, mm^{-1}	3.940	0.614
No. of unique data	3188	6487
No. of parameters, refined	185	442
GOF on F^2	1.060	1.049
$R1^a$ [$I > 2\sigma(I)$]	0.0428	0.0331
$R1^a$ (all data)	0.0588	0.0410
$wR2^b$ (all data)	0.0842	0.0894
Largest diff. peak and hole	0.795 and -0.718 e.Å^{-3}	0.247 and -0.263 e.Å^{-3}

$^a R1 = \frac{\sum ||Fo| - |Fc||}{\sum |Fo|}$; $^b wR2 = \sqrt{\frac{\sum [w(Fo^2 - Fc^2)^2]}{\sum [w(Fo^2)^2]}}$

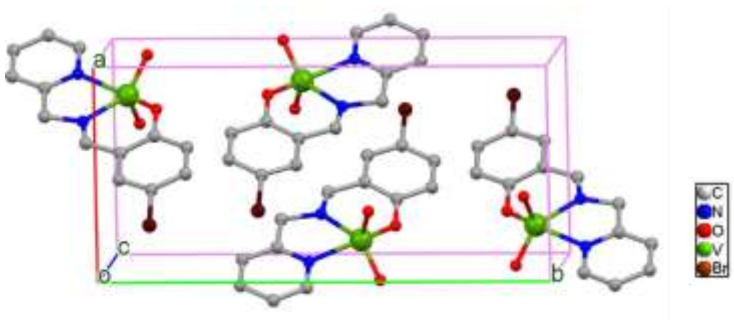

Fig. **S1**. Crystal packing diagram of complex **1A**, **$[V^VO_2L1]$** (**HL1** = 5-Bromosalicylidin-2-picolylimine). (H atoms are excluded for clarity).

Table S2. Selected bond lengths (Å) and bond angles (°) for complex **1A**, **$[V^VO_2L1]$** where (HL1 = 5-Bromosalicylidin-2-picolylimine).

Bond distances (Å)	Complex **1A**
V(1)-N(1)	2.115(3)
V(1)-N(2)	2.153(3)
V(1)-O(1)	1.900(2)
V(1)-O(2)	1.637(2)
V(1)-O(3)	1.620(2)
C(6)-N(2)	1.475(4)
C(7)-N(2)	1.293(4)
C(10)-Br(1)	1.901(3)
Bond angles (°)	
O(2)-V(1)-O(3)	109.32(12)
O(2)-V(1)-O(1)	96.48(11)
O(3)-V(1)-O(1)	105.70(11)
O(2)-V(1)-N(2)	141.84(11)
O(3)-V(1)-N(2)	107.32(11)
O(1)-V(1)-N(2)	83.52(10)
O(2)-V(1)-N(1)	89.13(11)
O(3)-V(1)-N(1)	99.41(11)
O(1)-V(1)-N(1)	150.68(10)
N(2)-V(1)-N(1)	74.66(10)

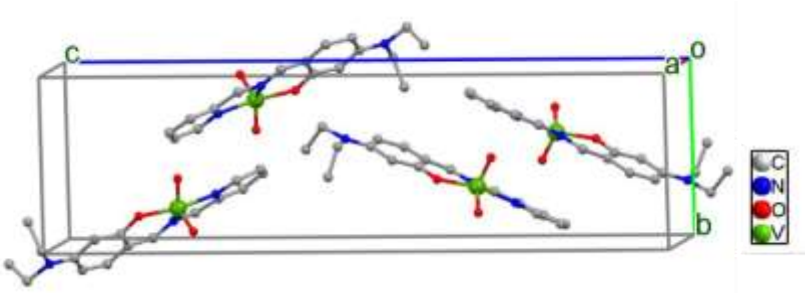

Fig. **S2**. Crystal packing diagram of complex **2A**, **[V^{V}O$_2$L2]** (HL2 = 4-Diethylaminosalicylidin-2-picolylimine). (H atoms are excluded for clarity).

Table S3. Selected bond lengths (Å) and bond angles (°) for complex **2A**, **[V^{V}O$_2$L2]** where (HL2 = 4-Diethylaminosalicylidin-2-picolylimine).

Bond distances (Å)	Molecule **A**	Bond distances (Å)	Molecule **B**
V(1A)-N(1A)	2.136(5)	V(1B)-N(1B)	2.140(5)
V(1A)-N(2A)	2.097(5)	V(1B)-N(2B)	2.127(4)
V(1A)-O(1A)	1.910(4)	V(1B)-O(1B)	1.888(3)
V(1A)-O(2A)	1.638(6)	V(1B)-O(2B)	1.609(6)
V(1A)-O(3A)	1.654(5)	V(1B)-O(3B)	1.595(6)
C(6A)-N(2A)	1.504(7)	C(6B)-N(2B)	1.434(8)
C(7A)-N(2A)	1.306(8)	C(7B)-N(2B)	1.316(8)
Bond angles (°)		Bond angles (°)	
O(2A)-V(1A)-O(3A)	110.7(2)	O(3B)-V(1B)-O(2B)	108.5(3)
O(2A)-V(1A)-O(1A)	103.7(2)	O(3B)-V(1B)-O(1B)	105.4(3)
O(3A)-V(1A)-O(1A)	98.0(2)	O(2B)-V(1B)-O(1B)	97.2(2)
O(2A)-V(1A)-N(2A)	110.7(2)	O(3B)-V(1B)-N(2B)	113.1(3)
O(3A)-V(1A)-N(2A)	136.7(3)	O(2B)-V(1B)-N(2B)	136.1(3)
O(1A)-V(1A)-N(2A)	83.97(17)	O(1B)-V(1B)-N(2B)	84.83(16)
O(2A)-V(1A)-N(1A)	96.2(2)	O(3B)-V(1B)-N(1B)	96.1(2)
O(3A)-V(1A)-N(1A)	88.1(2)	O(2B)-V(1B)-N(1B)	89.1(2)
O(1A)-V(1A)-N(1A)	155.49(18)	O(1B)-V(1B)-N(1B)	154.23(17)
N(2A)-V(1A)-N(1A)	75.58(18)	N(2B)-V(1B)-N(1B)	73.62(17)

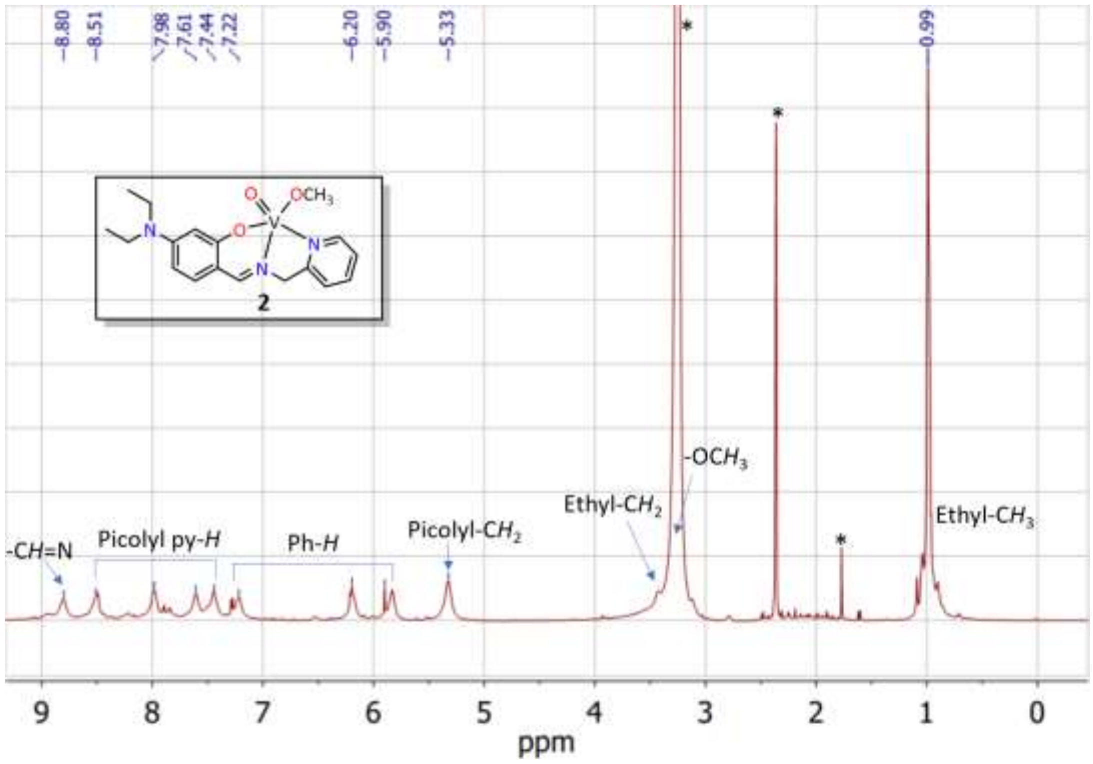

Fig. S3 ^{1}H NMR spectrum (in dmso-d_6) of complex **2**, **[$V^{IV}O(OCH_3)L2$]** (HL2 = 4-Diethylaminosalicylidin-2-picolylimine). Asterisks represent solvent impurities.

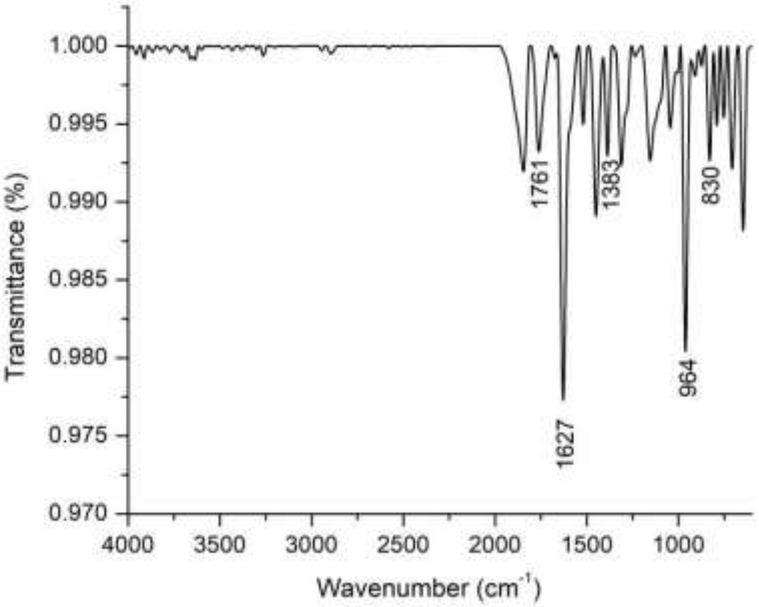

Fig. S4 IR spectrum of complex **1**, **[$V^{IV}O(H_2O)L1]NO_3$** (HL1 = 5-Bromosalicylidin-2-picolylimine).

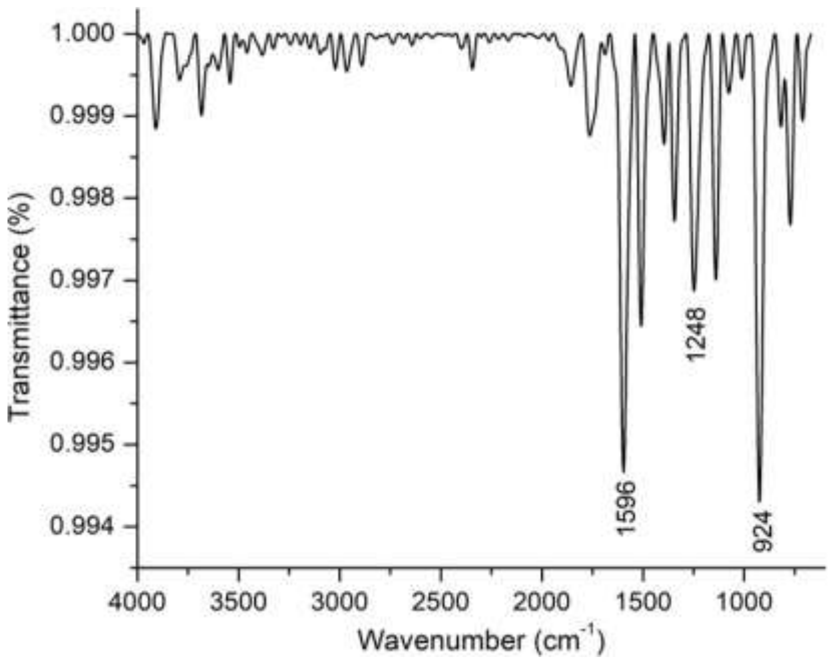

Fig. S5 IR spectrum of complex **2**, **[V^{IV}O(OCH$_3$)L2]** (HL2 = 4-Diethylaminosalicylidin-2-picolylimine).

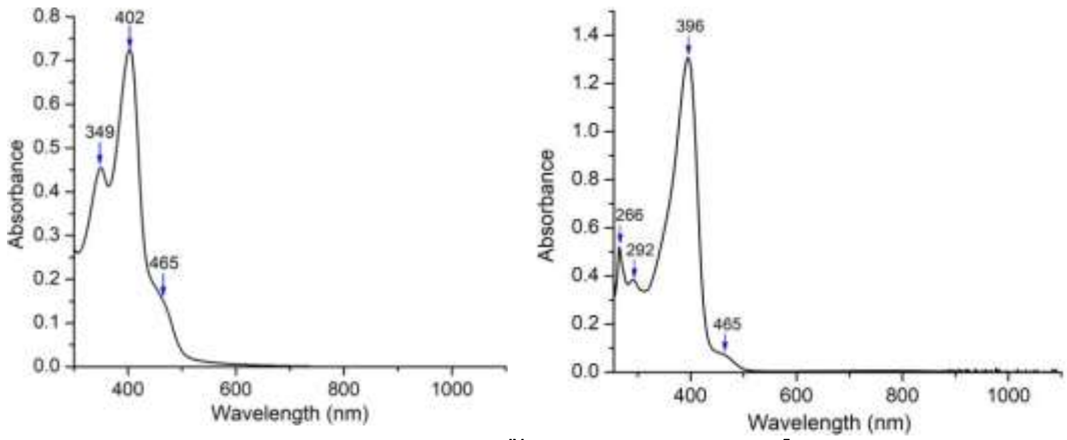

Fig. S6 UV-visible spectrum (left) of complex **2, [V^{IV}O(OCH$_3$)L2]** (5.0 × 10^{-5} M) in dichloromethane and, (right) complex **2A**, **[V^{V}O$_2$L2]** in dimethylformamide (5.0 × 10^{-5} M) at 298 K.

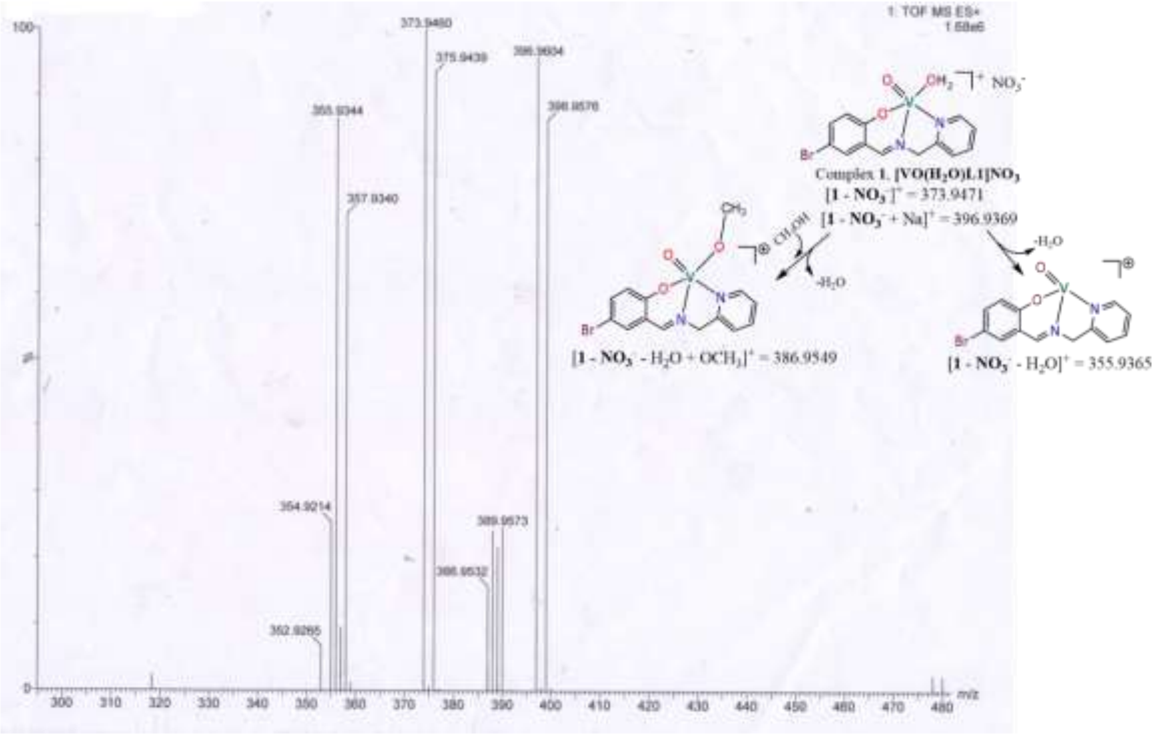

Fig. S7 ESI (+)-HRMS of complex **1**, **[V^{IV}O(H_2O)L1]NO_3** (HL1 = 5-Bromosalicylidin-2-picolylimine) and its fragments recorded in HRMS grade methanol.

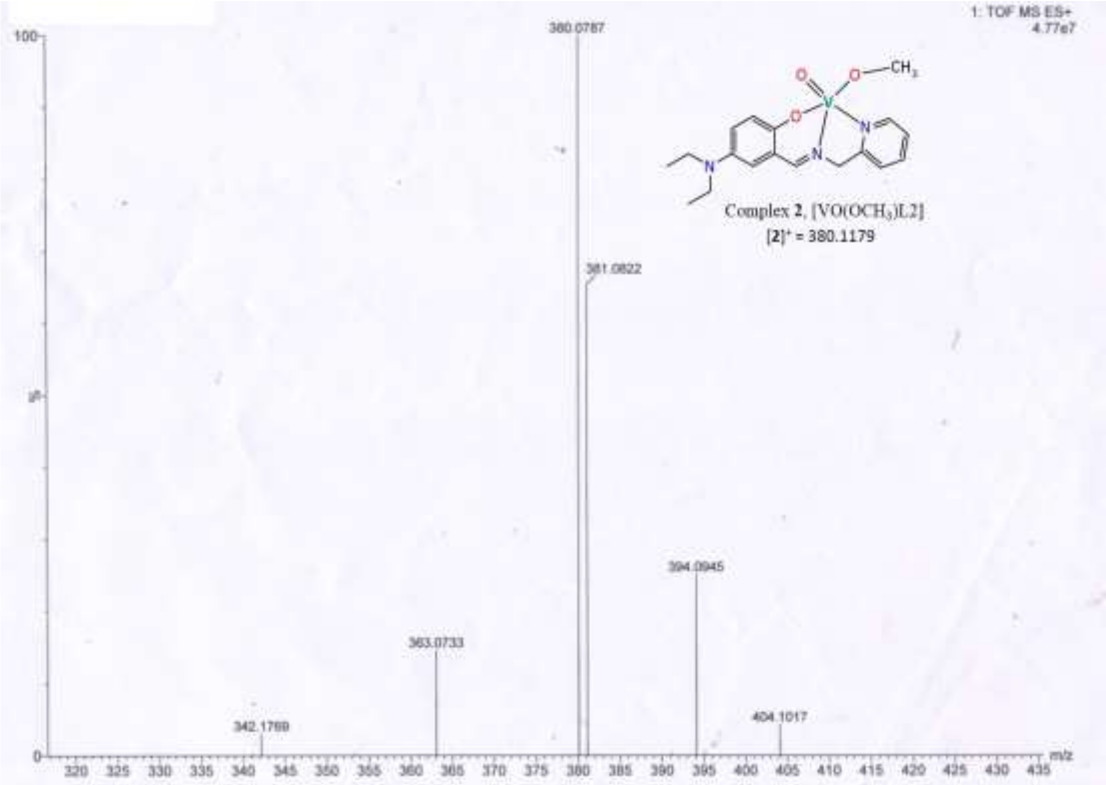

Fig. S8 ESI (+)-HRMS of complex **2**, **[V^{IV}O(OCH_3)L2]**, (HL2 = 4-Diethylaminosalicylidin-2-picolylimine) recorded in HRMS grade methanol.

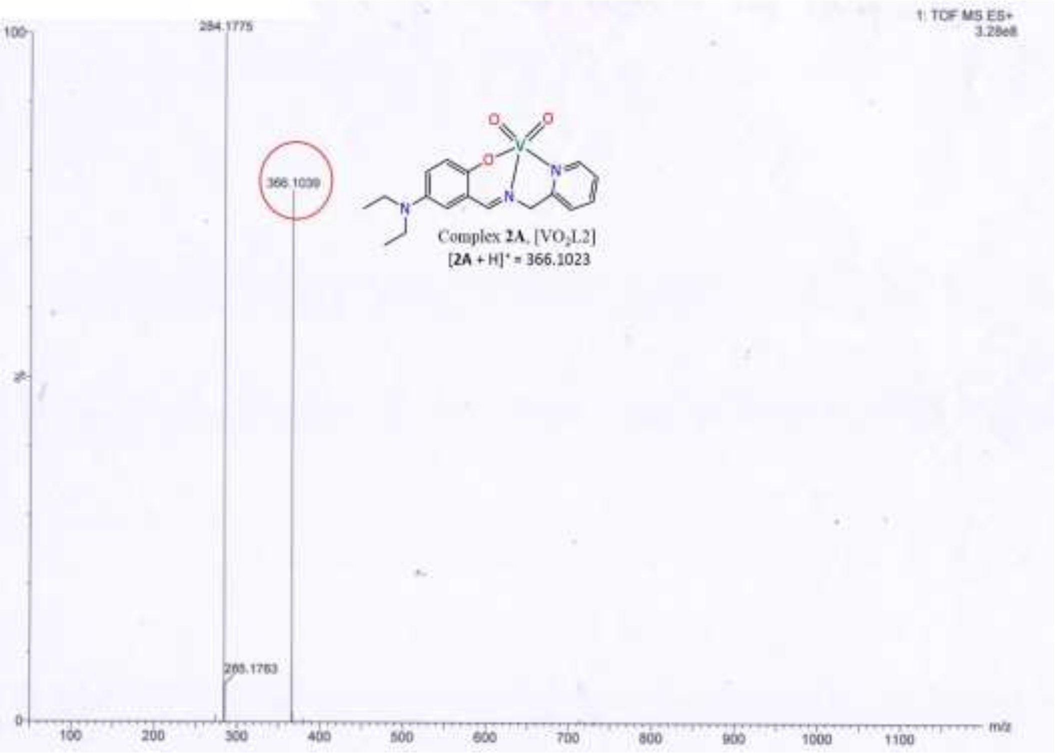

Fig. S9 ESI (+)-HRMS of complex **2A**, **[V^{V}O$_2$L2]** (HL2 = 4-Diethylaminosalicylidin-2-picolylimine) recorded by dissolving crystals of **2A** in HRMS grade methanol.

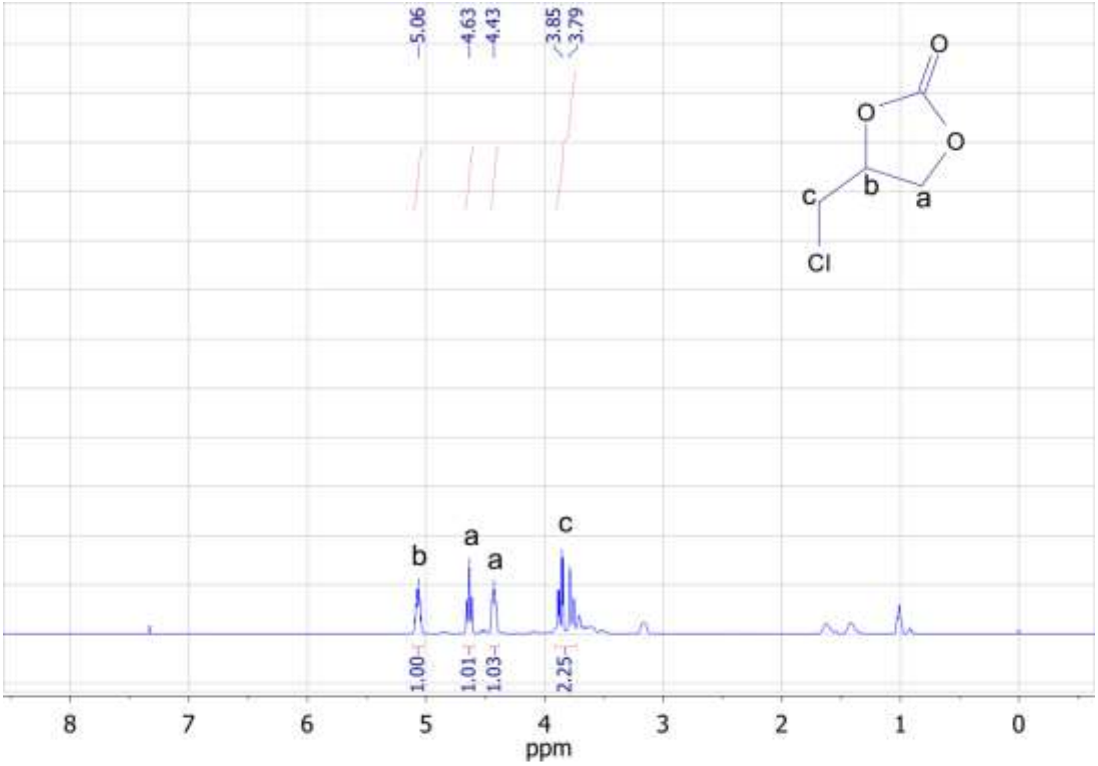

Fig. S10 ^{1}H NMR (400 MHz) spectrum of reaction mixture in $CDCl_3$. [Complex **1** (1 mol%); TBAB (2 mol%); temperature, 60 ^{o}C; time, 4 h; pressure (CO_2), 5 bar; conversion (%), 100%].

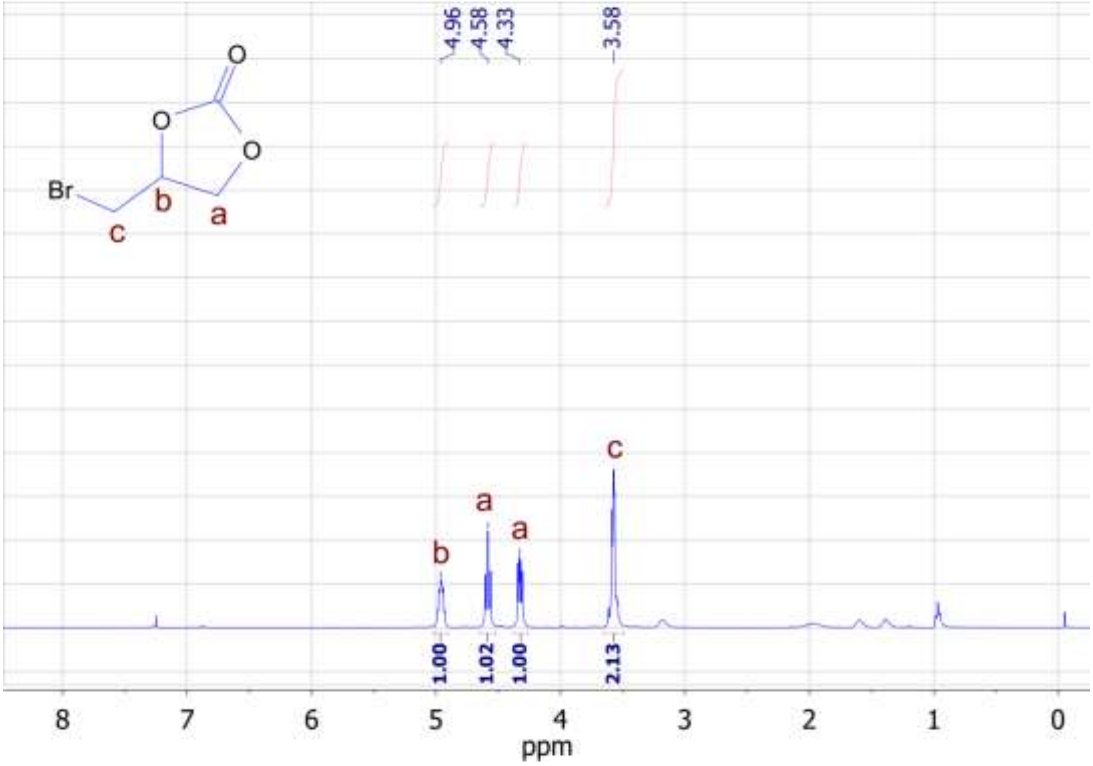

Fig. S11 ^{1}H NMR (400 MHz) spectrum of reaction mixture in $CDCl_3$. [Complex **1** (1 mol%); TBAB (2 mol%); temperature, 60 °C; time, 4 h; pressure (CO_2), 5 bar; conversion (%), 100%].

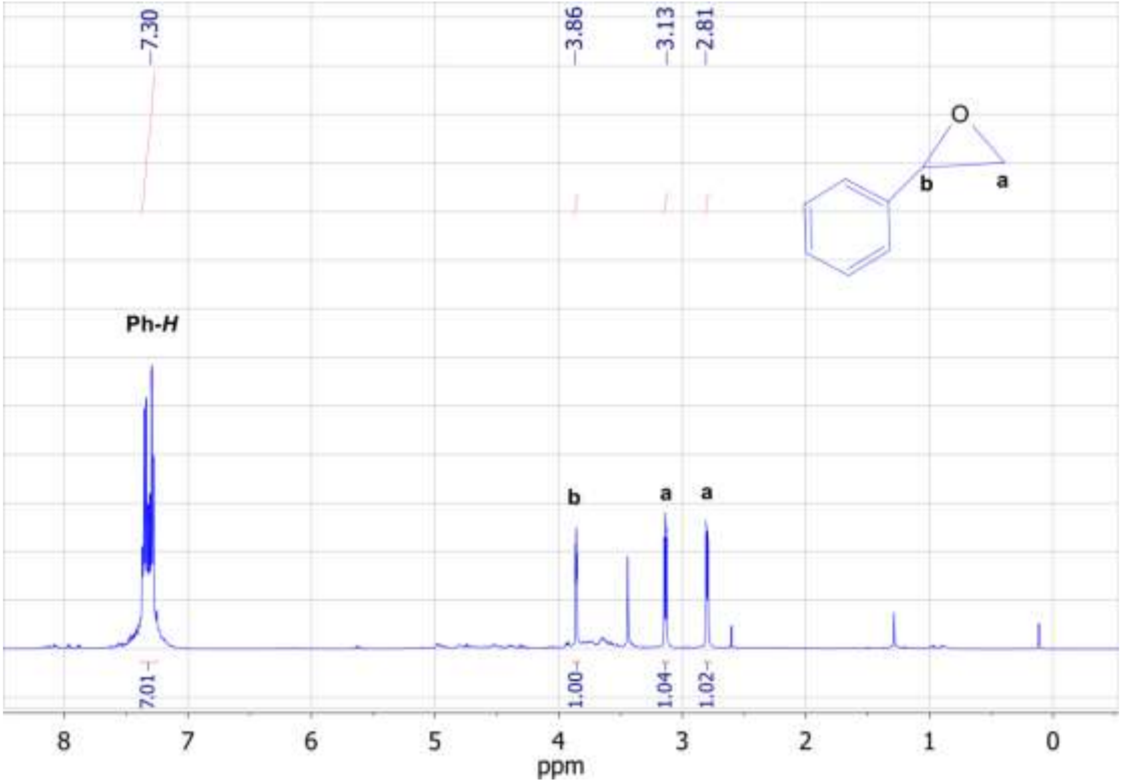

Fig. S12 ^{1}H NMR (500 MHz) spectrum of reaction mixture in $CDCl_3$. [Complex **1** (1 mol%); TBAB (0 mol%); temperature, 60 °C; time, 4 h; pressure (CO_2), 5 bar; conversion (%), 0].

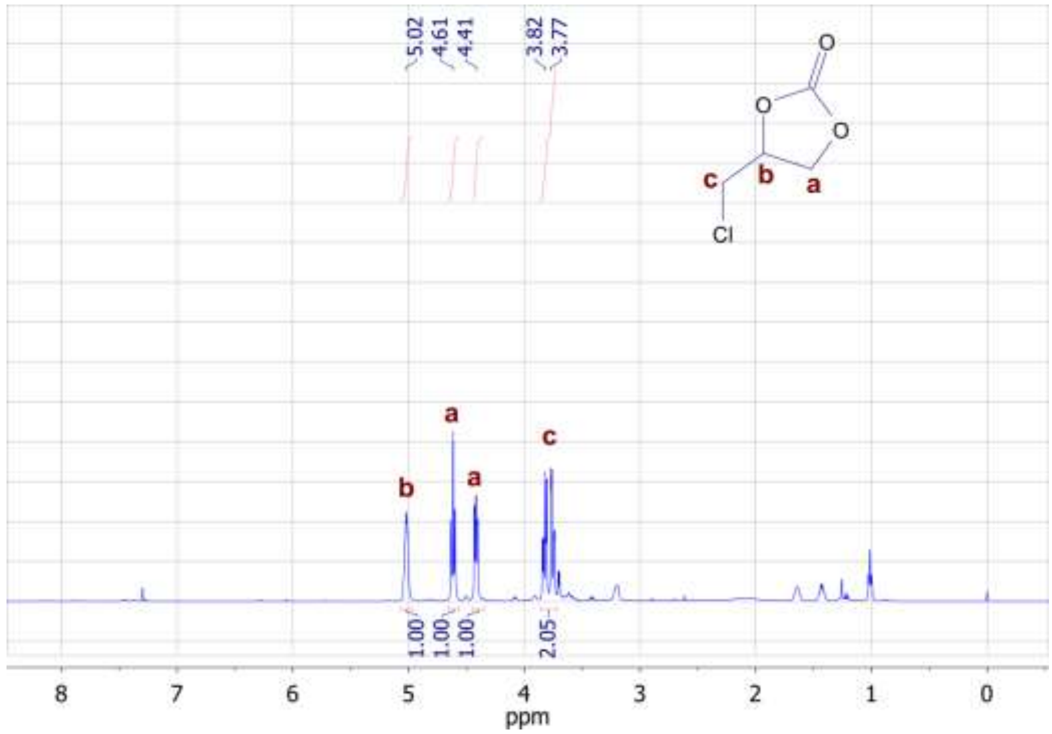

Fig. S13 ^{1}H NMR spectrum (500 MHz) of reaction mixture in $CDCl_3$. [Complex **2** (1 mol%); TBAB (2 mol%); temperature, 60 °C; time, 4 h; pressure (CO_2), 5 bar; conversion (%), 100%].

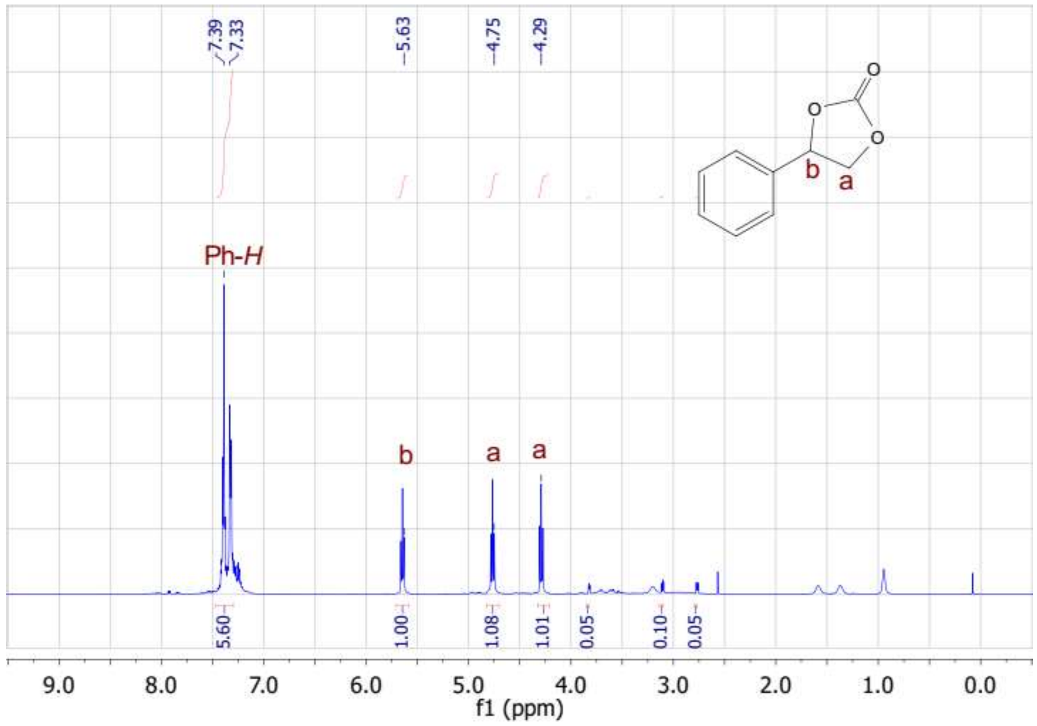

Fig. S14 ^{1}H NMR spectrum (500 MHz) of reaction mixture in $CDCl_3$. [Complex **1** (1 mol%); TBAB (2 mol%); temperature, 60 °C; time, 4 h; pressure (CO_2), 5 bar; conversion (%), 95 %].

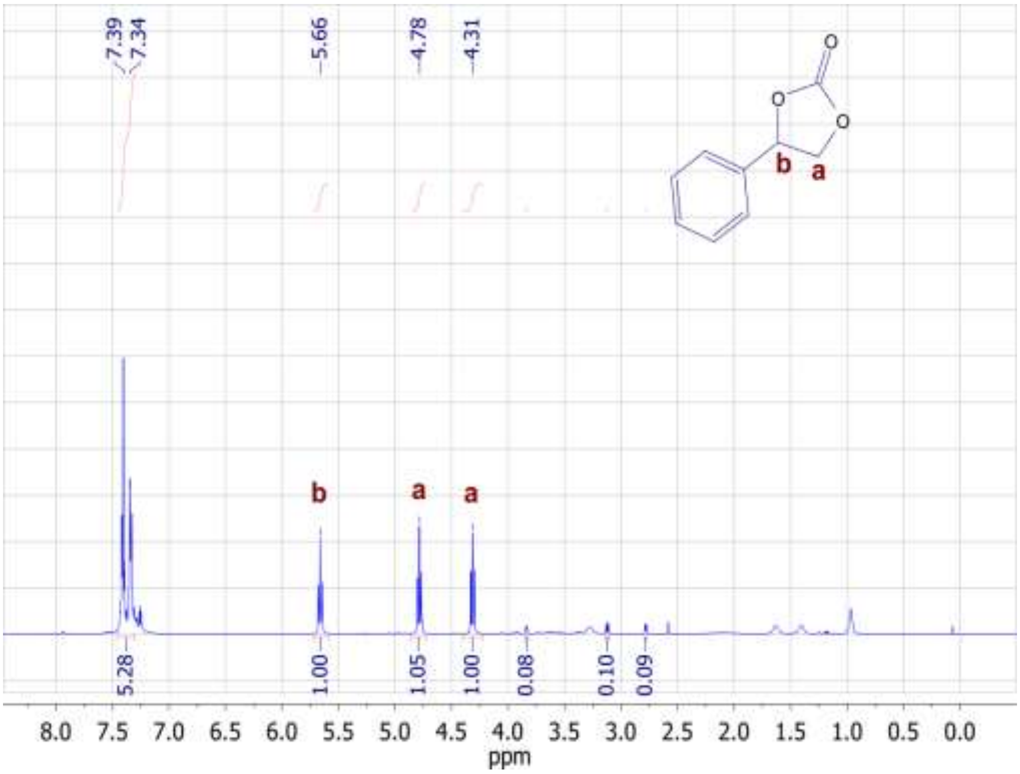

Fig. S15 ^{1}H NMR (500 MHz) spectrum of reaction mixture in $CDCl_3$. [Complex **2** (1 mol%); TBAB (2 mol%); temperature, 60 °C; time, 4 h; pressure (CO_2), 5 bar; conversion (%), 93%].

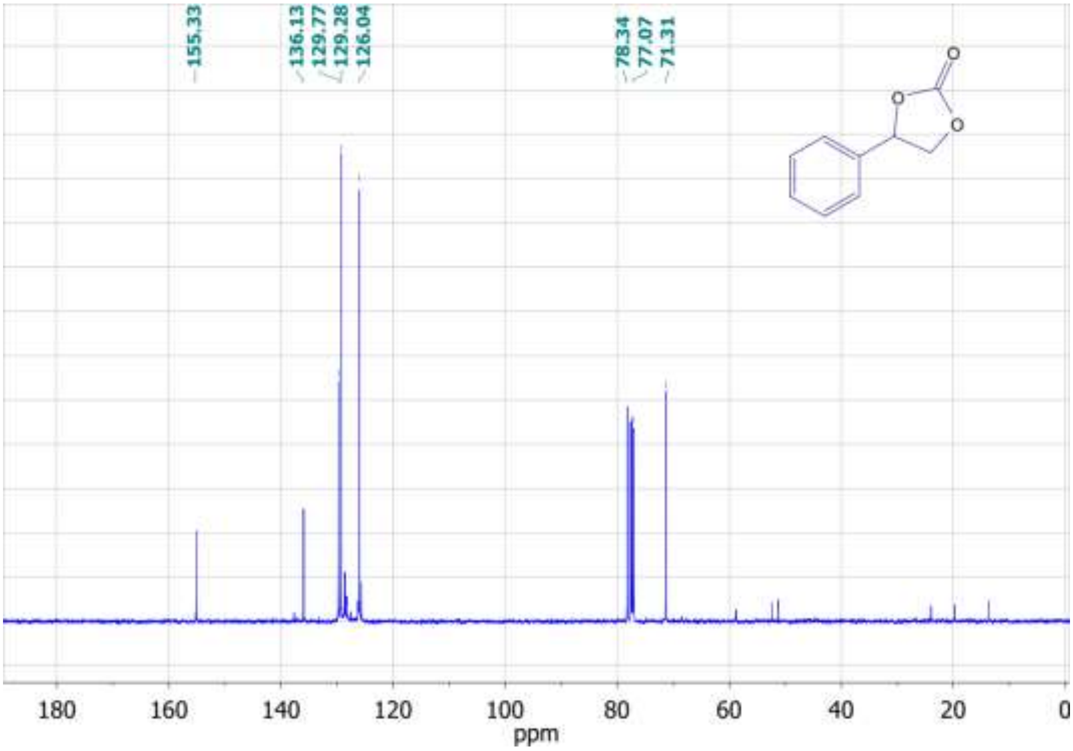

Fig. S16 ^{13}C {^{1}H} NMR (500 MHz) spectrum of **4-Phenyl-1,3-dioxola-2-one** in $CDCl_3$.

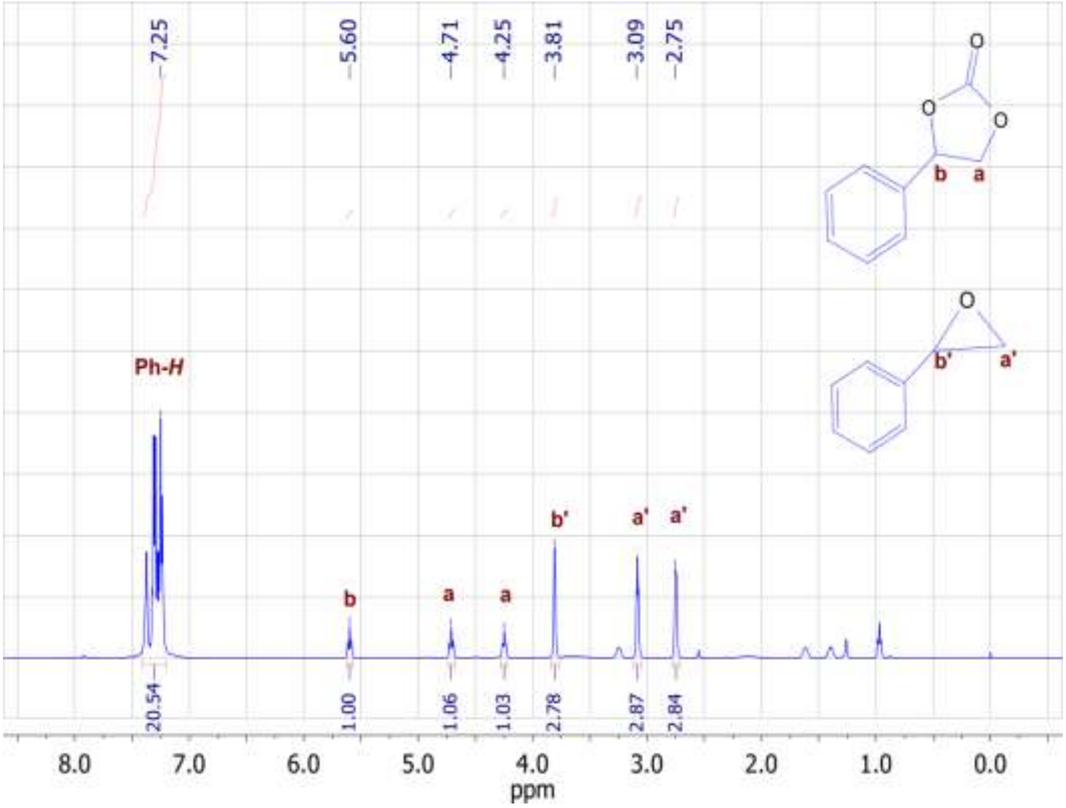

Fig. S17 ^{1}H NMR (500 MHz) spectrum of reaction mixture in $CDCl_3$. [Complex (0 mol%); TBAB (2 mol%); temperature, 60 °C; time, 4 h; pressure (CO_2), 5 bar; conversion (%), 26%].

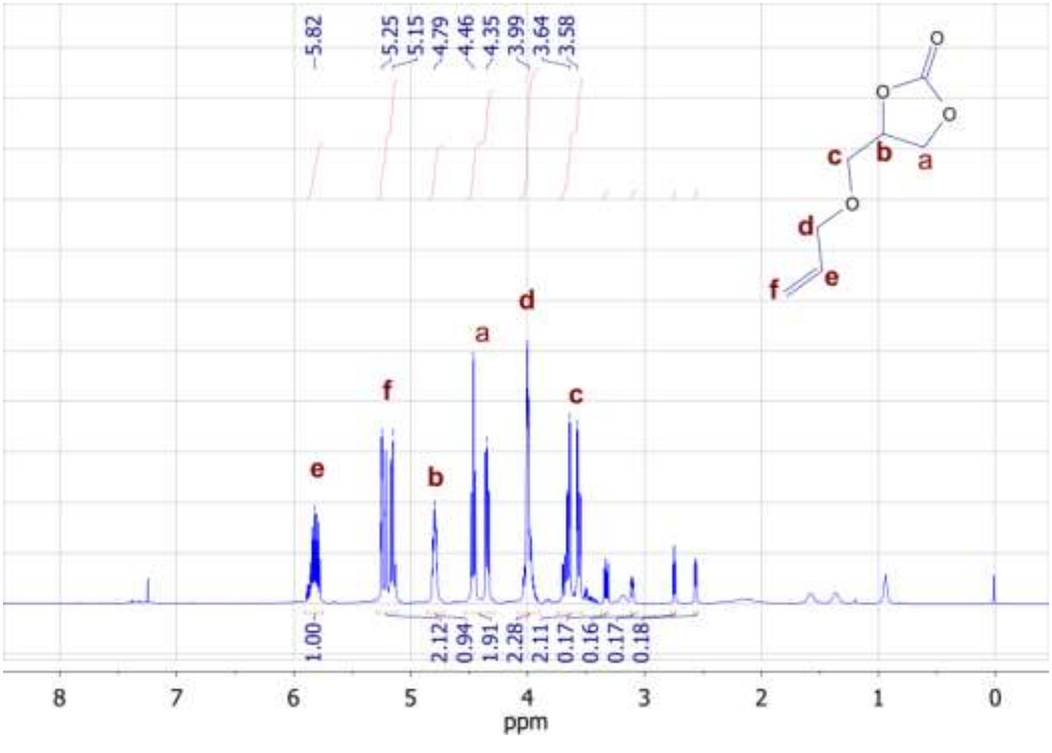

Fig. S18 ^{1}H NMR (500 MHz) spectrum of reaction mixture in $CDCl_3$. [Complex **1** (1 mol%); TBAB (2 mol%); temperature, 60 °C; time, 4 h; pressure (CO_2), 5 bar; conversion (%), 85%].

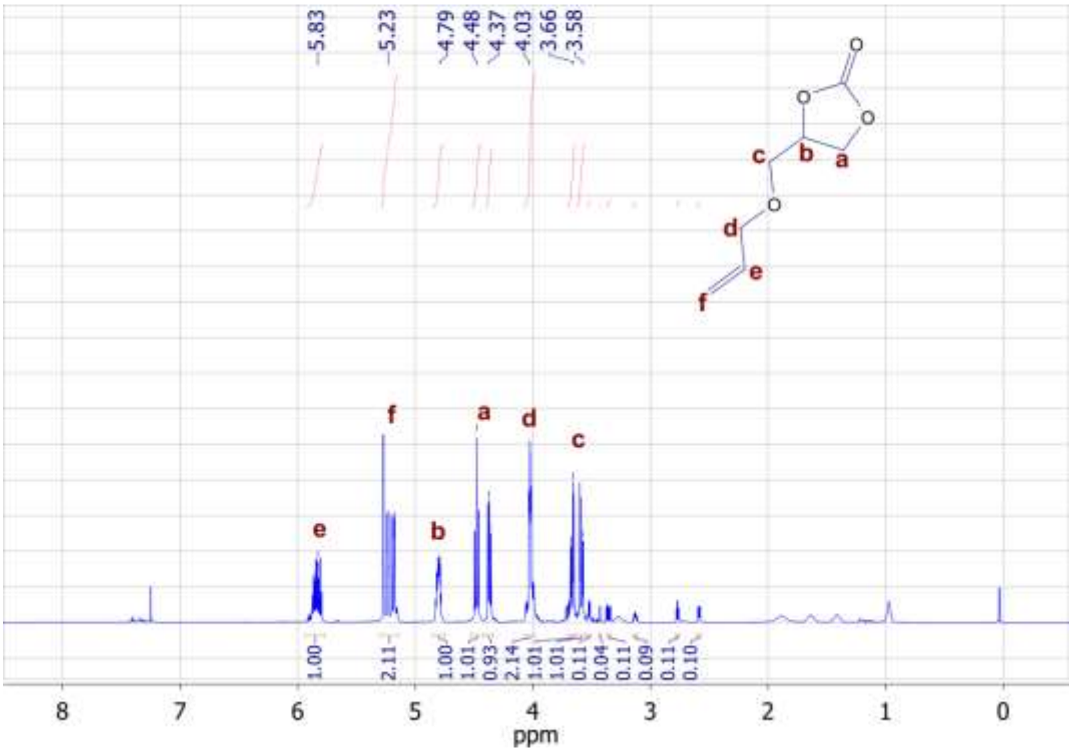

Fig. S19 ^{1}H NMR (500 MHz) spectrum of reaction mixture in $CDCl_3$. [Complex **2** (1 mol%); TBAB (2 mol%); temperature, 60 °C; time, 4 h; pressure (CO_2), 5 bar; conversion (%), 92%].

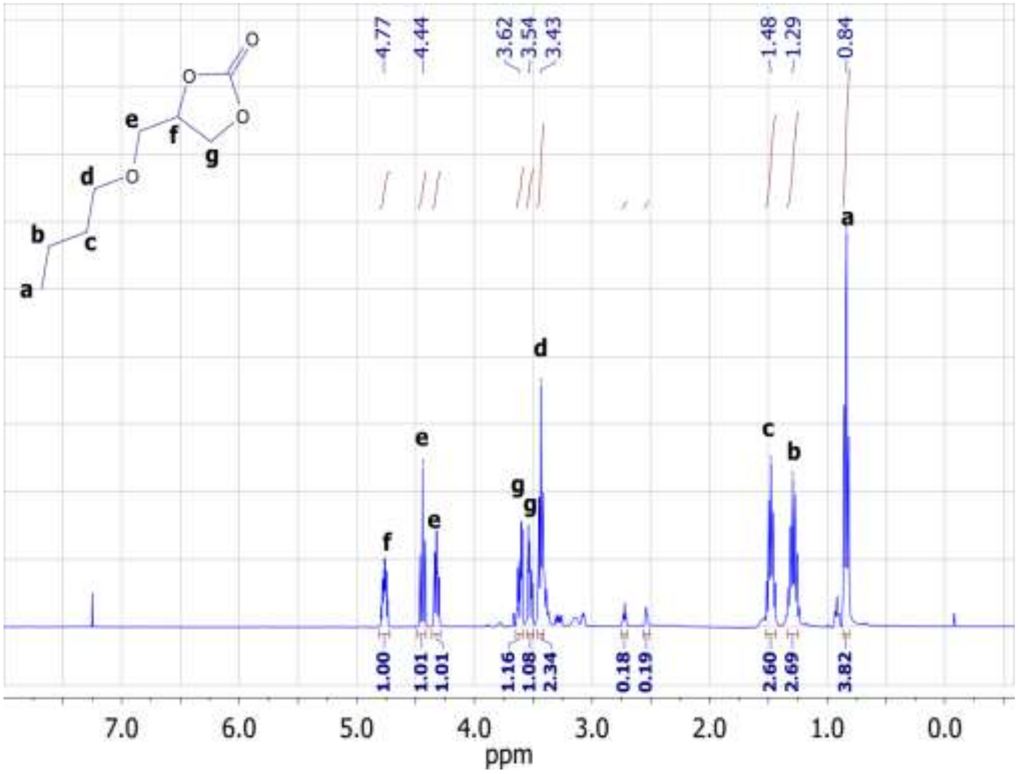

Fig. S20 ^{1}H NMR (400 MHz) Spectrum of reaction mixture in $CDCl_3$. [Complex **1** (1 mol%); TBAB (2 mol%); temperature, 60 °C; time, 4 h; pressure (CO_2), 5 bar; conversion (%), 85%].

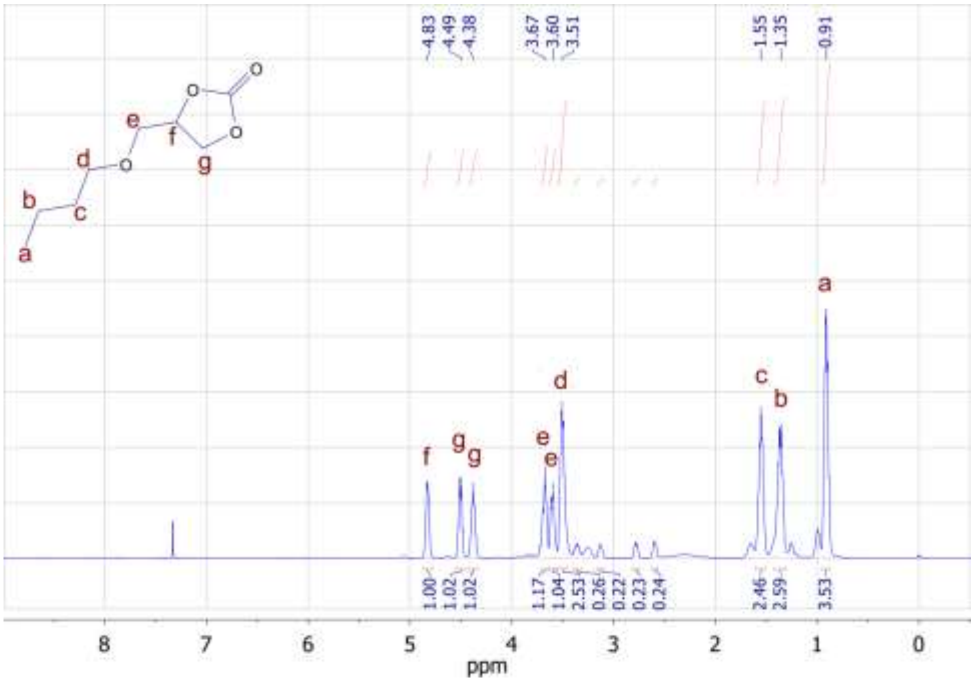

Fig. S21 ^{1}H NMR (500 MHz) Spectrum of reaction mixture in $CDCl_3$. [Complex **2** (1 mol%); TBAB (2 mol%); temperature, 60 °C; time, 4 h; pressure (CO_2), 5 bar; conversion (%), 82%].

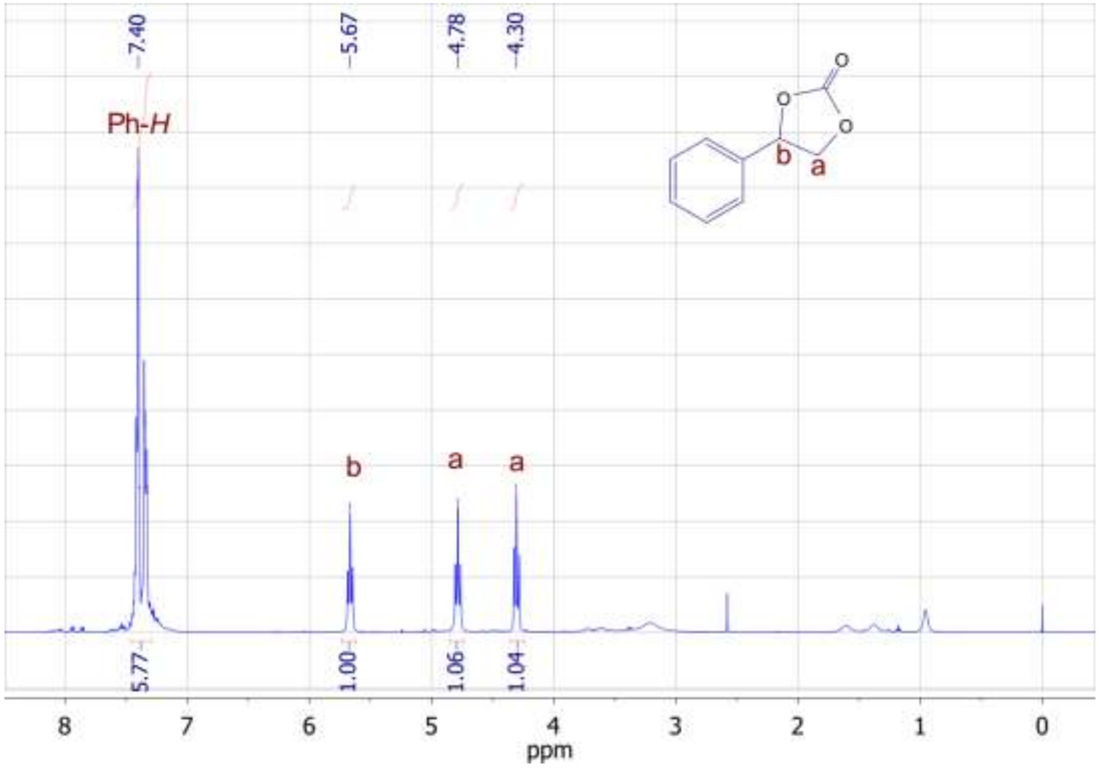

Fig. S22 ^{1}H NMR (400 MHz) spectrum of reaction mixture in $CDCl_3$. [Complex **1** (1 mol%); TBAB (2 mol%); temperature, 60 °C; time, 6 h; pressure (CO_2), 1 atm; conversion (%), 100%].

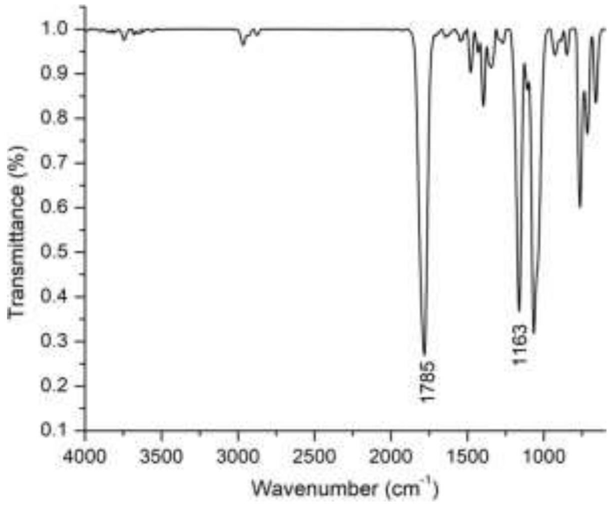

Fig. S23 IR spectrum of 4-(chloromethyl)-1,3-dioxolan-2-one.

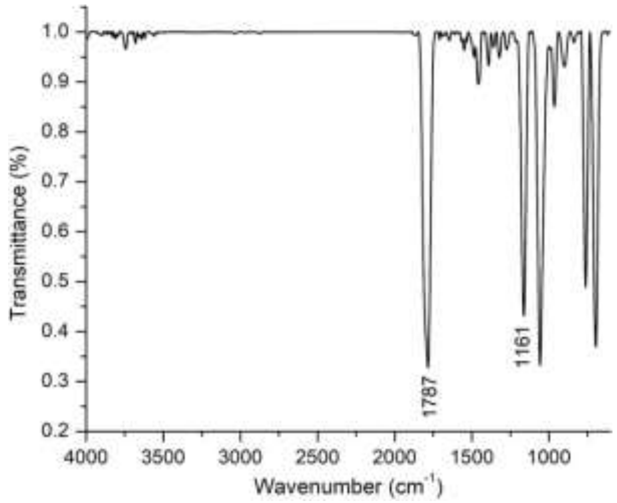

Fig. S24 IR spectrum of 4-phenyl-1,3-dioxolan-2-one.

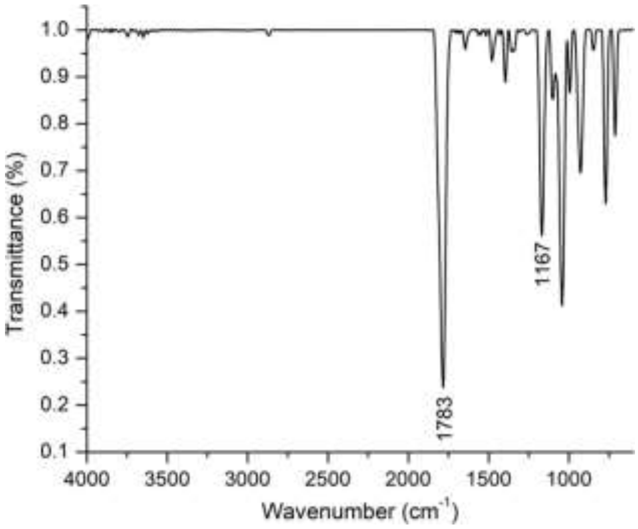

Fig. S25 IR spectrum of 4-((allyloxy)methyl)-1,3-dioxolan-2-one.

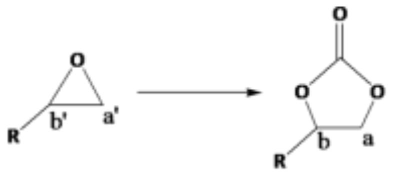

$$\% \text{ Conversion} = \frac{\text{Proton peak integration of } \mathbf{a}}{\text{Proton peak integration of } (\mathbf{a'} + \mathbf{a})} \times 100$$

Fig. S26 Calculation of % conversion of epoxide to cyclic carbonate from ^{1}H NMR spectrum.

Elemental (CHN) analysis for $C_{13}H_{12}N_2O_3BrV$, complex [**1** - NO_3^-]$^+$

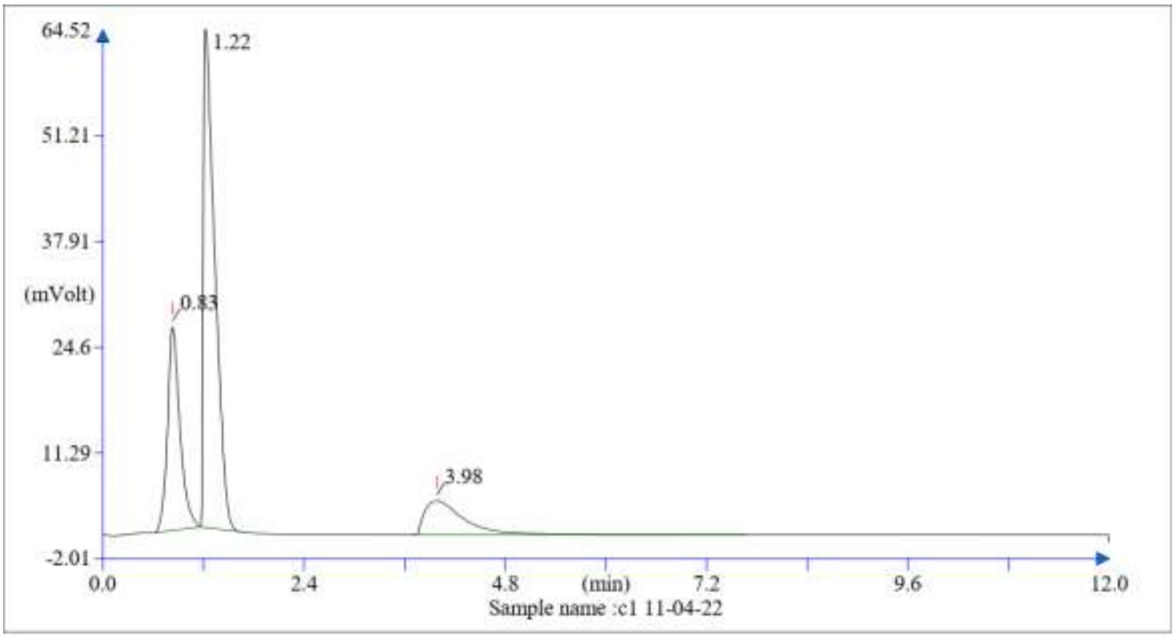

Ident. Number (#)	Component Name	Retention Time (min)	Area (.1*uV*sec)	Element %
1	Nitrogen	0.833	2650286	6.414
2	Carbon	1.217	6017466	42.123
3	Hydrogen	3.983	1389667	2.919
			10057420	51.455

GHV Heat value: 4458.105 (Kcal)
NHV Heat value: 4308.348 (Kcal)

Elemental (CHN) analysis for $C_{18}H_{23}N_3O_3V$, complex **2**

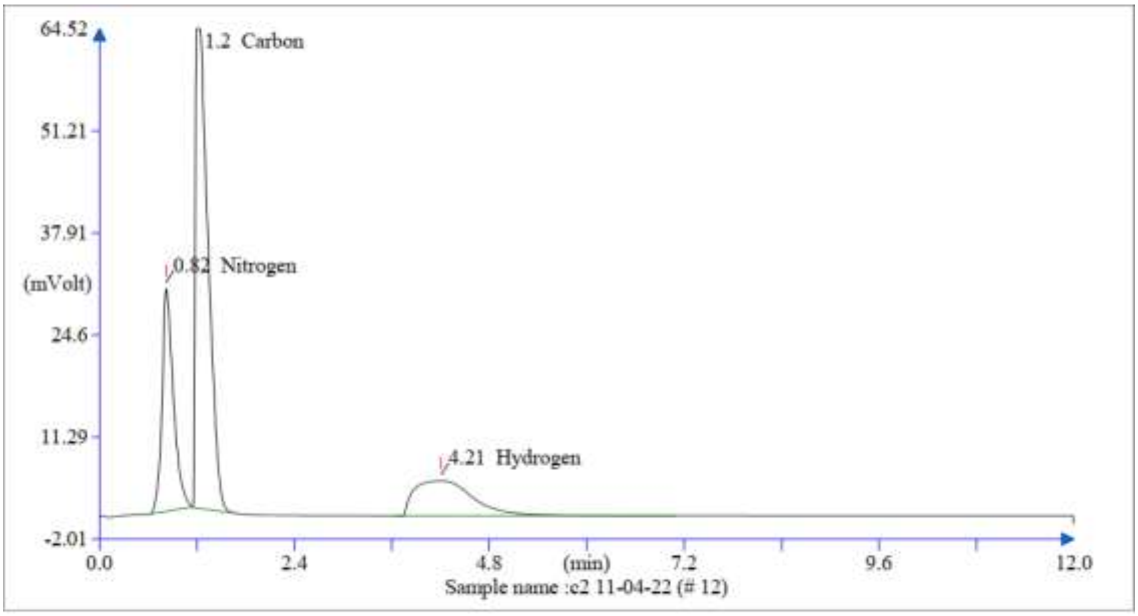

Ident. Number (#)	Component Name	Retention Time (min)	Area (.1*uV*sec)	Element %
1	Nitrogen	0.817	2838005	10.188
2	Carbon	1.200	7066484	56.460
3	Hydrogen	4.208	2313125	5.573
			12217610	72.221

GHV Heat value: 6546.792 (Kcal)
NHV Heat value: 6260.85 (Kcal)
